# Chemical mutagenesis and thermal selection of coral photosymbionts induce adaptation to heat stress with trait trade‐offs

**DOI:** 10.1111/eva.13586

**Published:** 2023-08-19

**Authors:** Hugo J. Scharfenstein, Carlos Alvarez‐Roa, Lesa M. Peplow, Patrick Buerger, Wing Yan Chan, Madeleine J. H. van Oppen

**Affiliations:** ^1^ School of BioSciences The University of Melbourne Parkville Victoria Australia; ^2^ Australian Institute of Marine Science Townsville Queensland Australia; ^3^ Applied BioSciences Macquarie University Sydney New South Wales Australia

**Keywords:** adaptation, climate change, corals, experimental evolution, mutagenesis, Symbiodiniaceae thermal tolerance

## Abstract

Despite the relevance of heat‐evolved microalgal endosymbionts to coral reef restoration, to date, few Symbiodiniaceae strains have been thermally enhanced via experimental evolution. Here, we investigated whether the thermal tolerance of Symbiodiniaceae can be increased through chemical mutagenesis followed by thermal selection. Strains of *Durusdinium trenchii*, *Fugacium kawagutii* and *Symbiodinium pilosum* were exposed to ethyl methanesulfonate to induce random mutagenesis, and then underwent thermal selection at high temperature (31/33°C). After 4.6–5 years of experimental evolution, the in vitro thermal tolerance of these strains was assessed via reciprocal transplant experiments to ambient (27°C) and elevated (31/35°C) temperatures. Growth, photosynthetic efficiency, oxidative stress and nutrient use were measured to compare thermal tolerance between strains. Heat‐evolved *D. trenchii*, *F. kawagutii* and *S. pilosum* strains all exhibited increased photosynthetic efficiency under thermal stress. However, trade‐offs in growth rates were observed for the heat‐evolved *D. trenchii* lineage at both ambient and elevated temperatures. Reduced phosphate and nitrate uptake rates in *F. kawagutii* and *S. pilosum* heat‐evolved lineages, respectively, suggest alterations in nutrition resource usage and allocation processes may have occurred. Increased phosphate uptake rates of the heat‐evolved *D. trenchii* strain indicate that experimental evolution resulted in further trade‐offs in this species. These findings deepen our understanding of the physiological responses of Symbiodiniaceae cultures to thermal selection and their capacity to adapt to elevated temperatures. The new heat‐evolved Symbiodiniaceae developed here may be beneficial for coral reef restoration efforts if their enhanced thermal tolerance can be conferred *in hospite*.

## 1 INTRODUCTION

Dinoflagellates in the family Symbiodiniaceae are widespread on tropical coral reefs. They commonly form endosymbioses with marine invertebrates spanning the phyla Cnidaria, Porifera, Mollusca and Foraminifera (Weber & Medina, 2012), while many species have also been recorded as free‐living in the water column and benthos (Coffroth et al., 2006; Fujise et al., 2021; Littman et al., 2008). Symbiodiniaceae provide vital photosynthate to their host, receiving host respiratory carbon and metabolic nitrogenous waste in return (reviewed by Davy et al., 2012). Scleractinian corals thrive in tropical oligotrophic waters thanks to this highly optimized nutrient exchange, relying on their Symbiodiniaceae communities to meet most of their energy requirements (Davies, 1984; Falkowski et al., 1984).

This symbiotic relationship is highly sensitive to environmental stressors, which may induce coral bleaching (i.e. the loss of Symbiodiniaceae from the coral tissues) and subsequent death if symbiosis cannot re‐establish (Brown, 1997). Higher than usual temperatures and/or irradiance are the primary causes of large‐scale coral bleaching events (Hoegh‐Guldberg, 1999). Thermal stress is hypothesized to induce excessive reactive oxygen species (ROS) production due to damage to the Symbiodiniaceae photosystems, resulting in detrimental oxidative stress in both partners and a host cellular cascade leading to the loss of the symbionts (Weis, 2008). An alternative theory is that a heat‐stress‐induced shift to host catabolism frees the symbiont of its nitrogen‐limited state, leading to increased symbiont growth and photosynthate retention (Rädecker et al., 2021; Wooldridge, 2013). This drives phosphorus starvation of the symbiont which alters the composition of its photosynthetic membranes and results in the malfunctioning of the photosystems, causing excessive ROS production by the symbionts (Wiedenmann et al., 2013).

Climate change‐driven increases in sea surface temperatures coupled with more frequent and severe marine heatwaves are posing a major threat to the survival of coral reefs (Hughes et al., 2017; Spalding & Brown, 2015). Whilst evolutionary adaptation may enable species to overcome stressful conditions and persist in the long term (Hoffmann & Sgró, 2011), the long sexual generation times of corals (3–5 years or longer) makes it likely that genetic adaptation occurs too slowly relative to the pace of rapid climate change (van Oppen et al., 2015). Indeed, the extensive coral loss observed during recent coral bleaching events worldwide, such as at Australia's Great Barrier Reef (Hughes et al., 2018, 2019), the Hawaiian Islands (Matsuda et al., 2020) and the Florida Keys (Fisch et al., 2019) may indicate that the adaptive capacity of coral thermal tolerance is being outpaced by the increase in severity, frequency and duration of summer heatwaves induced by climate change.

Assisted evolution refers to a series of bioengineering approaches that accelerate natural adaptative processes and the evolution of specific traits that will enhance coral bleaching resilience (van Oppen et al., 2015, 2017). Since coral bleaching resilience is partly dependent on the thermal tolerance of the intracellular Symbiodiniaceae communities (Berkelmans & van Oppen, 2006; Howells et al., 2012; Mieog et al., 2009; Silverstein et al., 2015), one assisted evolution strategy aims to accelerate the evolution of Symbiodiniaceae thermal tolerance through exposure to elevated temperatures in vitro (i.e. experimental evolution) (Chakravarti & van Oppen, 2018). The fast rate of asexual reproduction that can be achieved in Symbiodiniaceae cultures (2–20 days in vitro compared to 3.8–73.7 days *in hospite*; Wilkerson et al., 1988) means that adaptation to thermal stress can occur over a shorter timeframe than in the wild. Three long‐term evolutionary experiments (ranging from 1 to 4 years) have previously been carried out with Symbiodiniaceae strains that were cultivated under gradually increasing temperatures, which acted as a selective force (Chakravarti et al., 2017; Chakravarti & van Oppen, 2018; Huertas et al., 2011). Several heat‐evolved (i.e. thermally selected) strains of the species *Cladocopium proliferum* (formerly *C. goreaui/Cladocopium* C1^acro^; Butler et al., 2023) were found to display faster growth rates and higher photosynthetic efficiency than their wild‐type counterparts under elevated temperatures. After reintroduction into aposymbiotic coral larvae, three of these selected strains were found to improve the thermal bleaching resilience of the host (Buerger et al., 2020). Additionally, one of these strains was tested in symbiosis with juvenile corals and showed it can enhance their survival under elevated temperatures (Quigley & van Oppen, 2022). These findings demonstrate that in vitro thermal tolerance can be conferred upon the coral host and that experimental evolution can be a highly relevant strategy to produce enhanced coral stock for coral reef restoration.

The family Symbiodiniaceae comprise at least 15 genera and genus‐level lineages (LaJeunesse et al., 2018; Pochon & LaJeunesse, 2021), of which the genera *Cladocopium*, *Durusdinium* and *Symbiodinium* are the most‐commonly found symbionts in corals (Baker, 2003). Other genera tend to occur at background levels, such as representatives of the genus *Fugacium* found at <0.1% and 5% relative abundance in the corals *P. lutea* and *D. gravida*, respectively (Qin et al., 2019; Teschima et al., 2019). The Symbiodiniaceae family comprises many species across its 15 genera, displaying a high biodiversity which is often reflected functionally, meaning the physiological performance of the coral host can differ based on the identity of its symbionts (Mieog et al., 2009). Natural variation occurs in Symbiodiniaceae thermal tolerance, and fitness trade‐offs with thermal tolerance exist. For instance, thermotolerant members of the genus *Durusdinium* have been reported to increase the thermal bleaching resilience of their coral hosts by as much as 1–2°C compared to *Cladocopium* symbionts (Berkelmans & van Oppen, 2006; Silverstein et al., 2017), though at the cost of slower photosynthate translocation and coral growth at ambient temperatures than members of the genus *Cladocopium* (Cantin et al., 2009; Jones & Berkelmans, 2010; Little et al., 2004). Several Symbiodiniaceae species may coexist within a coral host, forming communities dominated by a single or handful of species or strains depending on the life stage of the coral (Silverstein et al., 2012). Many Symbiodiniaceae species have a narrow host range (Smith et al., 2017; Thomas et al., 2014), while some ‘generalist’ Symbiodiniaceae are found across many different host taxa (Fabina et al., 2012; LaJeunesse, 2002). Hence, expanding the taxonomic diversity of heat‐evolved Symbiodiniaceae is important to broaden the target host range for reintroduction as part of assisted evolution efforts.

In microalgal research, a growing number of studies have used chemical mutagenesis followed by thermal selection to improve the thermal tolerance of microalgae and cyanobacteria (Chou et al., 2019; Ong et al., 2010; Sachdeva et al., 2016; Tillich et al., 2012). Mutagenesis is used to create genetic diversity for thermal selection to act upon, increasing the likelihood of a beneficial trait for thermal tolerance arising in mutagen‐treated lineages. Exposure to N‐methyl‐N′‐nitro‐N‐nitrosoguanidine (Chou et al., 2019) and ethyl methanesulfonate (Ong et al., 2010; Sachdeva et al., 2016) followed by screening under elevated temperatures ranging from 25 to 50°C has successfully been used to increase the thermal tolerance of *Chlorella* spp. These promising results have led us to consider the incorporation of chemical mutagenesis as part of the experimental evolution efforts of Symbiodiniaceae.

In this study, cultures belonging to the genera *Durusdinium*, *Fugacium* and *Symbiodinium* were subjected to chemical mutagenesis followed by thermal selection at elevated temperatures. Improvements in the in vitro thermal tolerance of these heat‐evolved strains, characterized here as an increase in growth, photochemical efficiency and/or reduction in ROS levels under thermal stress, were subsequently assessed via reciprocal transplant experiments to ambient and elevated temperatures.

## 2 MATERIALS AND METHODS

### 2.1 Organisms and mutagenesis

Three strains belonging to the Symbiodiniaceae species *Durusdinium trenchii*, *Fugacium kawagutii* and *Symbiodinium pilosum* were isolated from scleractinian corals collected in the central and southern Great Barrier Reef (Table 1). The *F. kawagutii* strain (SCF089.01) was obtained from the University of Technology Sydney (Suggett et al., 2015), whilst the *D. trenchii* (SCF086.01) and *S. pilosum* (SCF004.01) strains were isolated at the Australian Institute of Marine Science as described by Chakravarti and van Oppen (2018).

**TABLE 1:** List of Symbiodiniaceae species used in this study.

Symbiodiniaceae species investigated (ITS2 type)	Isolated from	Number of MT lineages generated	Thermal selection temperature (°C)	MT lineages alive after^a^			EE start date	RTE start date	Culture transfers till RTE	Lineages tested in RTE	Strain
				1 week	3 months	4 years					
*S. pilosum* (A2)	*Galaxea* sp.	32	33	16	16	5	Apr 2017	Oct 2021	21	SCF 004.01	WT
			34	16	16	0				SCF 004.01.01	MT
*D. trenchii* (D1)	*Porites lobata*	32	31	16	16	2	Apr 2017	Apr 2022	23	SCF 086.01	WT
			32	16	0	0				SCF 086.01.04	MT
*F. kawagutii* (F1)	*Pocillopora damicornis*	32	33	16	16	5	Apr 2017	Oct 2021	21	SCF 089.01	WT
			34	16	16	0				SCF 089.01	MT

*Note*: The mutagen‐treated (MT) and the wild‐type (WT) strains were derived from the same mother culture. The number of culture transfers elapsed between the onset of the experimental evolution (EE) and reciprocal transplant experiment (RTE) are indicated for the MT strains. Following recovery from chemical mutagenesis, the 32 MT cultures were split equally between two elevated temperatures. All cultures originated from the Great Barrier Reef, Australia.

^a^ The number of MT lineages alive relative to the time of EMS exposure (thermal selection began 1‐week following chemical mutagenesis).

In April 2017, sub‐cultures from all three strains underwent chemical mutagenesis following the methodology established by Doan and Obbard (2012). For each strain, replicate 30 mL cultures at 15,000 cells/mL (*n* = 32 per strain) were exposed to ethyl methanesulfonate (EMS) at a concentration of 100 mM (see Figure S1 for experimental design). The cultures were incubated with EMS at 27°C for 1 h and agitated using an orbital shaker, after which they were centrifuged at 4000 *g* for 5 min. The supernatant was discarded and 35 mL of a 10% (w/v) sodium thiosulfate solution was added to the pelleted cultures to remove any residual EMS. The cultures were then centrifuged (4000 *g* × 5 min) and the supernatant was discarded. This step was repeated with fresh sodium thiosulfate twice more. The resulting mutagen‐treated (MT) cultures were resuspended in 0.2 μm filter‐sterilized seawater with added Daigo's IMK Medium (398–01333, Nihon Pharmaceutical Co. Ltd., Tokyo, JP; see Table S1 for recipe) and left to recover at 27°C for 1 week. For the remainder of this study, all cultures were maintained in the IMK culture medium.

The MT *D. trenchii* cultures were subsequently moved to 31 and 32°C, whilst the MT *S. pilosum* and *F. kawagutii* cultures were transferred to 33 and 34°C (*n* = 16 per strain/temperature). These temperatures were chosen because they were previously found to induce decreased photosynthetic performances in the respective strains (Figure S2). The MT strains were maintained at the highest temperature they survived at (i.e. 31°C for *D. trenchii*, 33°C for *S. pilosum* and *F. kawagutii*), to facilitate adaptation to the elevated temperatures. The wild‐type (WT) cultures that were not exposed to the mutagen were kept at 27°C.

The WT and MT strains were sub‐cultured in fresh IMK medium approximately every 11 weeks. The strains were grown in 25 cm^2^ cell culture flasks (0.2 μm vent cap; CLS430639, Sigma‐Aldrich) and maintained at their respective temperatures in environmental chambers (Steridium, Brendale, AU). The cultures followed a 14 h:10 h light:dark cycle and were exposed to a light intensity of 60 ± 10 μmol/m^2^/s (Sylvania F15W/T8/865 light tubes; 0000947, Sylvania).

### 2.2 Reciprocal transplant experiment design

After 4.6–5 years at elevated temperature (see Table 1 for durations), two reciprocal transplant experiments (RTEs) to ambient and elevated temperatures were carried out (i.e. a temperature inducing decreased photosynthetic efficiency, serving as a proxy for thermal stress; Warner et al., 1999) to assess the in vitro thermal tolerance of one MT lineage relative to one WT lineage. To meet the high biomass requirements for the RTEs, we selected the fastest‐growing MT lineage from each species since the growth of the surviving MT lineages had been severely impacted. The RTEs followed the methodology previously established by Chakravarti and van Oppen (2018) and Buerger et al. (2020). These consisted of comparing the physiological performances of WT and MT strains derived from the same mother culture at both ambient and elevated temperatures. The *D. trenchii* strains were exposed to an elevated temperature of 31°C, whilst the elevated temperature (35°C) for the *S. pilosum* and *F. kawagutii* strains was guided by the results from a pilot study in which the photosynthetic efficiency of these strains was monitored over 24–26 days at temperatures ranging 33–35°C (Figure S3).

Biomass from the WT and MT strains was grown 2 months prior to the RTEs. Once sufficient biomass was obtained (~4 × 10^8^ cells per strain), half of the biomass from each strain was transferred to the elevated temperature (31/35°C) and the remaining half to the ambient temperature (27°C). The stock cultures were ramped up/down to their final temperatures at a rate of 2°C a day and reached their target temperature simultaneously. Cultures were left to acclimate for 2 weeks before starting measurements to ensure long‐term thermal adaptation rather than acclimation was being measured (Chakravarti et al., 2017).

Following acclimation, roughly 75% of the culture medium was discarded from the stock cultures and replaced with fresh IMK medium. Biomass loss was minimal due to the benthic behaviour of the Symbiodiniaceae cells which remained settled on the flask bottom and walls. The cells were then resuspended and fresh IMK medium was inoculated at a cell density of 200,000 cells/mL, at a final culture volume of 20 mL. A total of 50 flasks (biological replicate cultures) were prepared per strain per temperature treatment. Sampling was carried out twice weekly for a duration of 32–33 days. At each sampling timepoint, five flasks per strain per temperature treatment were removed from the experiment for physiological analyses (see Figure S1). This sampling strategy was selected due to the invasive nature of the sampling, which involved the resuspension of the cultures using cell scrapers. The thermal tolerance of the strains was assessed through comparisons of growth and photosynthetic efficiency measurements, oxidative stress accumulation and nutrient use (for each assay, *n* = 5 per strain/temperature/timepoint). Assays and replication levels are listed in Table 2.

**TABLE 2:** Physiological traits assessed during the reciprocal transplant experiments.

Trait measured	Parameter	Description	Unit	Biological replication	Technical replication
Growth	Cell density Growth rate	Cell density within cultures Maximum growth rate	Cells/mL/d	5	2
Photosynthetic efficiency	*F*_v_/*F*_m_ *Q*_m_	Maximum quantum yield of PSII Maximum excitation pressure of PSII	Relative units Relative units	5	3
Oxidative stress	Intracellular ROS	Production of ROS within Symbiodiniaceae cells	Relative units	5	1
	Extracellular ROS	Accumulation of ROS within culture medium	ExROS/cell	5	4
Nutrient usage	${\mathrm{NO}}_{3}^{-}$ concentration	Nitrate uptake	μmol/L	5	1
	${\mathrm{NH}}_{4}^{+}$ concentration	Ammonium uptake	μmol/L	5	1
	${\mathrm{PO}}_{4}^{-3}$ concentration	Phosphate uptake	μmol/L	5	1

*Note*: Biological replication levels indicated are for each strain at each temperature at each timepoint the assay was carried out. Technical replication levels are for each sample.

### 2.3 Growth

The growth performance of each strain was assessed by measuring the cell densities of the cultures at each timepoint. Once the cells were resuspended through cell scraping followed by pipetting, a 970 μL aliquot was taken from each culture. The samples were fixed in 20 μL of 25% glutaraldehyde solution (G5882; Sigma‐Aldrich) and 10 μL of 1% Pluronic F‐68 (24040‐032; Thermo Fisher Scientific) was added. The samples were briefly vortexed, after which they were sonicated for a duration of 5 s at 40% amplitude (VCX 130 Vibra‐Cell Processor) to dissociate cell clusters. For both *S. pilosum* WT and MT strains, the cultures displayed significant clumping meaning a longer sonication duration (20 s) was used. The samples were processed using a flow cytometer (BD Accuri™ C6 Plus Flow Cytometer, BD Biosciences) at a speed of 35 μL/min (50 μL of sample processed) and excited at 488 nm. Autofluorescent events emitting at 675 ± 25 nm (chlorophyll fluorescence) were gated for Symbiodiniaceae cells and quantified to obtain the cell density. Each sample was measured twice (two technical replicates) and then averaged to obtain the final cell count.

Growth rates were calculated from the exponential growth phases of each strain under each temperature treatment according to the following equation:

$$\mu =\left[\ln\left(C_{1}\right)/\ln\left(C_{0}\right)\right]/\text{∆}t$$

where C_1_/C_0_ are the cell densities at the end/start of the exponential phase and ∆*t* is the duration of the exponential phase in days. To accurately identify exponential phases, linear models were fitted to log‐transformed growth curves in R using the package *growthrates* (version 0.8.4; Hall et al., 2014). Timepoints that deviated from the linear models delineated the limits of the exponential phase.

### 2.4 Photochemical efficiency

The photochemical efficiency of each strain was assessed using an imaging pulse‐amplitude modulation chlorophyll fluorometer (Maxi version IMAGING PAM M‐Series, Walz). In the dark, the flasks were removed from the environmental chambers and placed under the IMAGING PAM. The maximum quantum yield of photosystem II (PSII) of dark‐adapted cultures (*F*_v_/*F*_m_ = [*F*_m_−*F*_0_]/*F*_m_) was measured at the end of their dark cycle. The cultures were subsequently light‐adapted for 5 min using the actinic light of the IMAGING PAM and the effective quantum yield of PSII ($F_{v}^{\prime }/F_{m}^{\prime }=\left[F_{m}^{\prime }-F_{0}^{\prime }\right]/F_{m}^{\prime }$) was measured. The maximum excitation pressure over PSII ($Q_{m}=1–\left[\left(F_{v}^{\prime }/F_{m}^{\prime }\right)/\left(F_{v}/F_{m}\right)\right]$) was calculated according to Iglesias‐Prieto et al. (2004). Three areas of interest (technical replicates) were measured for each flask. The following IMAGING PAM parameters were used: light intensity = 3 for *D. trenchii* and *S. pilosum*/2 for *F. kawagutii*, gain = 1, damping = 2, actinic light intensity = 2.

### 2.5 Oxidative stress

Oxidative stress was assessed every third sampling timepoint (roughly every 1.5 weeks) by measuring the amount of net intracellular and extracellular ROS. Intracellular ROS measurements were carried out by modifying the methodology established by (Buerger et al., 2023). Each culture was sampled to obtain a 1 mL aliquot normalized to 500,000 cells/mL. The normalized samples were pelleted through centrifugation (5000 g × 5 min) and resuspended in 1 mL of fresh IMK culture medium. This procedure was repeated once more to wash the samples. The samples were then split in half: (1) a 500 μL aliquot that served as an unstained control and (2) a 500 μL aliquot that was stained with CellROX Green Reagent (C10444; Thermo Fisher Scientific) for intracellular ROS detection at a final concentration of 5 μM. The samples were subsequently incubated in the dark for 1 h at room temperature, after which they were briefly sonicated (3–5 s) before being measured through flow cytometry (BD FACSVerse, BD Biosciences). Samples were processed at a speed of 120 μL/min and excited at 488 nm. Autofluorescent events emitting at 700 ± 54 nm were used to gate for Symbiodiniaceae cells. Single cells were then gated using a FSC‐Height/FSC‐Area plot. The fluorescence of the CellROX Green Reagent was measured by quantifying the median fluorescent intensity (MFI) of the events emitting at 527 ± 37 nm (threshold set at 10,000 singlets). The MFI of a sample was obtained by subtracting the MFI of the unstained control aliquot (MFI_UC_) from the MFI of the corresponding CellROX Green stained aliquot (MFI_CRG_).

The accumulation of ROS in the culture medium (extracellular ROS) was measured according to the methodology described by Buerger et al. (2020) and Chakravarti et al. (2017). A 1 mL aliquot was taken from each culture and pelleted through centrifugation (14,000 *g* × 5 min). The supernatant from one sample was transferred to four wells (*n* = 4 technical replicates, 230 μL supernatant/well) in a black clear‐bottomed 96‐well plate (3603; Sigma‐Aldrich). For each plate, fresh IMK culture medium, serving as a blank, was transferred to an additional four wells, whilst four wells were left empty. In the dark, CellROX Orange (C10443; Thermo Fisher Scientific) was added to each well at a final concentration of 5 μM. The samples were incubated in the dark at 37°C for 30 min, after which the absorbance of each well was measured with a microplate reader (Synergy H4) at 540‐nm excitation and 565‐nm emission. The average absorbance of blank and empty wells was calculated, which was subtracted from the blank and empty well absorbance for each 96‐well plate to determine the dispersion of blank/empty well absorbance. To standardize across measurements from different days and plates, the sum of these values obtained from all the plates was then subtracted from the absorbance of the sample to obtain an adjusted absorbance (see calculations in database S1 from Buerger et al., 2020). The median adjusted absorbance from the four technical replicates was calculated for each sample and normalized to cell density.

### 2.6 Macronutrient concentrations of the culture medium

The nutrient content in the culture medium was analysed over the course of the RTEs to gain a better understanding of whether nutrient uptake may differ between Symbiodiniaceae strains. A 10 mL sample of homogenized culture was taken from each flask and centrifuged at 3000 *g* for 5 min. The supernatant samples were filtered through 0.45 μm Minisart Syringe Filters (1655‐K; Sartorius) to remove any residual organic matter from the culture medium. The filtered samples were then frozen and stored at −20°C before being sent for nutrient content analysis (ammonium—${\mathrm{NH}}_{4}^{+}$, phosphate—${\mathrm{PO}}_{4}^{-3}$ and nitrate—${\mathrm{NO}}_{3}^{-}$) at the Analytical Technology Laboratory of the Australian Institute of Marine Science. The samples were processed using a segmented flow analyzer (AA3 HR AutoAnalyzer; Seal Analytical) following the manufacturer's guidelines.

A modified approach from Orefice et al. (2019) was used to calculate nitrate and phosphate uptake rates for each strain under each temperature treatment. The following equation was used:

$$\mathrm{Nu}=\left[\left(N_{1}-N_{0}\right)/\left(C_{1}-C_{0}\right)\right]/\text{∆}t$$

where Nu is the nutrient uptake rate (μmol/cells/day), N_1_/C_1_ are nutrient concentrations (μM)/cell densities (cells/mL) at the end of the exponential growth phase, N_0_/C_0_ are nutrient concentrations/cell densities at the start of the RTE and ∆*t* is the number of days elapsed between both timepoints. Mean nitrate and phosphate concentrations for each timepoint were used.

### 2.7 Statistical analyses

Growth, photochemical efficiency, ROS levels and nutrient uptake rates were tested for significant differences between strains and temperatures within each symbiont species. Statistical analyses were performed in R by carrying out generalized linear models (GLMs, package *stats* version 4.3.0.). Model assumptions were verified using the package *DHARMa* (version 0.4.6).

We examined the three‐way interactions of temperature (levels: ambient and elevated), strain (levels: WT and MT) and time (day, as a categorical variable) on the cell density yields and photochemical efficiency (*F*_v_/*F*_m_ and *Q*_m_) using GLMs. Gamma and quasibinomial distributions were used for the cell density and photochemical efficiency GLMs, respectively. The effect of two‐way interactions of temperature and strain on growth rates and nutrient uptake rates were analysed using GLMs with Gaussian and quasibinomial distributions, respectively. Delta values of intracellular and extracellular ROS levels (i.e. differences in intracellular/extracellular ROS levels between the elevated and ambient temperatures) were calculated for each strain and analysed using linear models looking at the two‐way interactions between strain and time. Analyses of deviance for the GLM fits were carried out to analyse the main effect of temperature and/or strain on the response variable.

Estimated marginal means were calculated for each physiological trait using the package *emmeans* (version 1.8.1‐1) to carry out a *post hoc* analysis of differences between strains and temperatures. The estimated marginal means enabled us to conduct pairwise comparisons between strains at each temperature (strain contrast: WT vs. MT strain) and for each strain between temperatures (temperature contrast: ambient vs. elevated) to test for significant effects of strain and temperature on the physiological trait measured. For time series data (i.e. cell density yields, *F*_v_/*F*_m_ and *Q*_m_), the estimated marginal means were grouped into early (measurements from timepoints 1–3), mid (timepoints 4–6) and late (timepoints 7–10) experimental phase. This grouping was carried out to facilitate the analysis due to the variability of the physiological responses over the course of the RTEs. For the ROS analyses, pairwise comparisons were carried out between strains (strain contrast only) at each timepoint rather than within each experimental phase.

## 3 RESULTS

### 3.1 Enhanced photochemical efficiency at 31°C comes at a cost of reduced growth at both ambient and elevated temperatures in MT *D. trenchii*

**FIGURE 1:**
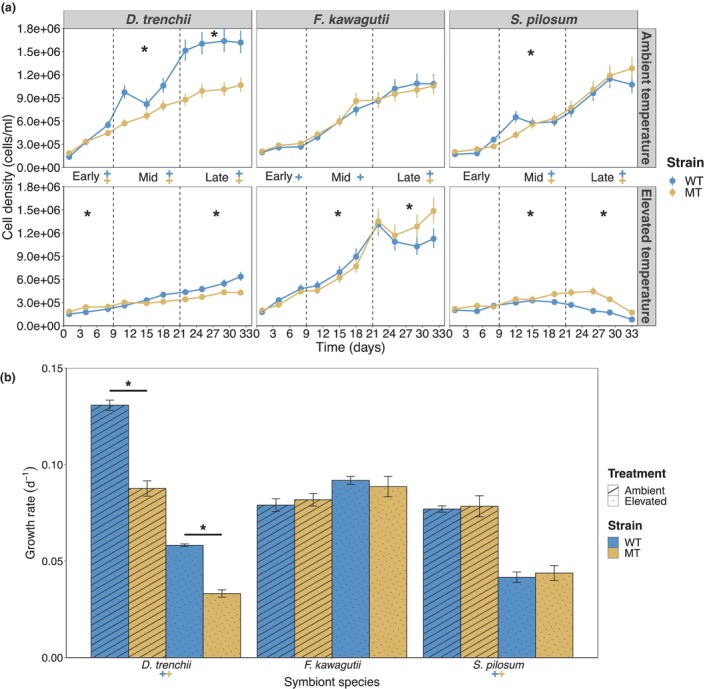
Growth performances of *Durusdinium trenchii, Fugacium kawagutii* and *Symbiodinium pilosum* cultures. Cell densities (a) and growth rates (b) of mutagen‐treated (MT) and wild‐type (WT) strains at ambient (27°C) and elevated (31°C for *D. trenchii*, 35°C for *F. kawagutii* and *S. pilosum*) temperatures. Significant differences (*p* < 0.01) are indicated for each experimental phase (as defined in Section 2, a). *, differences between both strains at the indicated temperature; +, differences between temperatures for the WT strain; +, differences between temperatures for the MT strain. Error bars represent 95% confidence intervals (a) and 1 standard error (b). *n* = 5 for each strain at each timepoint.

Growth of both the WT and MT *D. trenchii* strains was considerably inhibited by exposure to the elevated temperature (Figure 1; see Table 3 for a summary of analyses of deviance of GLM fits). Growth rates were 55% and 62% lower for the WT and MT strains, respectively, at 31°C than at 27°C (*p* < 0.001; Figure 1b; see Table S2 for a summary of pairwise comparisons). Further, the MT strain grew significantly less than its WT counterpart at 27 and 31°C (Figure 1a), displaying growth rates 33 and 43% lower, respectively (*p* < 0.001; Figure 1b).

**TABLE 3:** Outputs from analyses of deviance for the Generalized Linear Model fits that examined the effects of strain and temperature on cell density yields, growth rates, photochemical efficiency (*F*_v_/*F*_m_ and *Q*_m_), intracellular and extracellular ROS levels and nutrient (nitrate and phosphate) uptake rates on cultures from *Durusdinium trenchii, Fugacium kawagutii* and *Symbiodinium pilosum*.

Response	Interactions	df	df resid.	*D. Trenchii*		*F. Kawagutii*		*S. pilosum*	
				*F* ratio	Prob > *F*	*F* ratio	Prob > *F*	*F* ratio	Prob > *F*
Cell density yields	Strain	1	198	480.9	<0.001	3.2	0.074	40.6	<0.001
	Temperature	1	197	3850.0	<0.001	101.0	<0.001	2370.3	<0.001
	Time	9	188	420.3	<0.001	440.9	<0.001	145.0	<0.001
	Strain × Temperature	1	187	90.4	<0.001	0.7	0.41	53.5	<0.001
	Strain × Time	9	178	22.9	<0.001	1.1	0.387	13.0	<0.001
	Temperature × Time	9	169	56.5	<0.001	8.5	<0.001	173.6	<0.001
	Strain × Temperature × Time	9	160	5.7	<0.001	4.0	<0.001	7.0	<0.001
Growth rates	Strain	1	18	173.0	<0.001	0.0	0.957	0.2	0.635
	Temperature	1	17	600.0	<0.001	7.2	0.02	88.5	<0.001
	Strain × Temperature	1	16	12.3	0.003	0.7	0.423	0.0	0.9157
*F*_v_/*F*_m_	Strain	1	198	8.5	0.004	71.1	<0.001	0.5	0.462
	Temperature	1	197	540.5	<0.001	8550.7	<0.001	4857.3	<0.001
	Time	9	188	57.0	<0.001	138.7	<0.001	73.4	<0.001
	Strain × Temperature	1	187	469.5	<0.001	27.2	<0.001	50.2	<0.001
	Strain × Time	9	178	10.6	<0.001	10.3	<0.001	45.0	<0.001
	Temperature × Time	9	169	60.9	<0.001	96.4	<0.001	55.7	<0.001
	Strain × Temperature × Time	9	160	5.0	<0.001	8.8	<0.001	64.5	<0.001
*Q*_m_	Strain	1	198	2.0	0.162	31.9	<0.001	18.5	<0.001
	Temperature	1	197	1.0	0.33	645.8	<0.001	288.3	<0.001
	Time	9	188	46.0	<0.001	102.5	<0.001	54.1	<0.001
	Strain × Temperature	1	187	45.3	<0.001	138.4	<0.001	23.8	<0.001
	Strain × Time	9	178	11.0	<0.001	1.6	0.126	14.7	<0.001
	Temperature × Time	9	169	10.5	<0.001	37.0	<0.001	75.7	<0.001
	Strain × Temperature × Time	9	160	10.2	<0.001	1.5	0.169	3.8	<0.001
Delta InROS	Strain	1	38	17.43	<0.001	0.00	1.000	0.53	0.470
	Time	3	35	16.97	<0.001	27.86	<0.001	25.12	<0.001
	Strain × Time	3	32	3.11	0.04	0.89	0.456	0.24	0.8673
Delta ExROS	Strain	1	38	7.88	0.008	0.11	0.747	13.47	<0.001
	Time	3	35	50.70	<0.001	10.61	<0.001	48.19	<0.001
	Strain × Time	3	32	14.03	<0.001	0.99	0.411	9.19	<0.001
Nitrate uptake	Strain	1	18	1.6	0.2244	0.07	0.791	1.9	0.19
	Temperature	1	17	70.5	<0.001	44.52	<0.001	0.9	0.36
	Strain × Temperature	1	16	1.5	0.244	5.83	0.028	6.8	0.02
Phosphate uptake	Strain	1	18	62.33	<0.001	138.34	<0.001	2.99	0.103
	Temperature	1	17	89.27	<0.001	99.18	<0.001	0.15	0.71
	Strain × Temperature	1	16	13.22	0.002	96.36	<0.001	1.75	0.204

Abbreviation: df, degrees of freedom.

**FIGURE 2:**
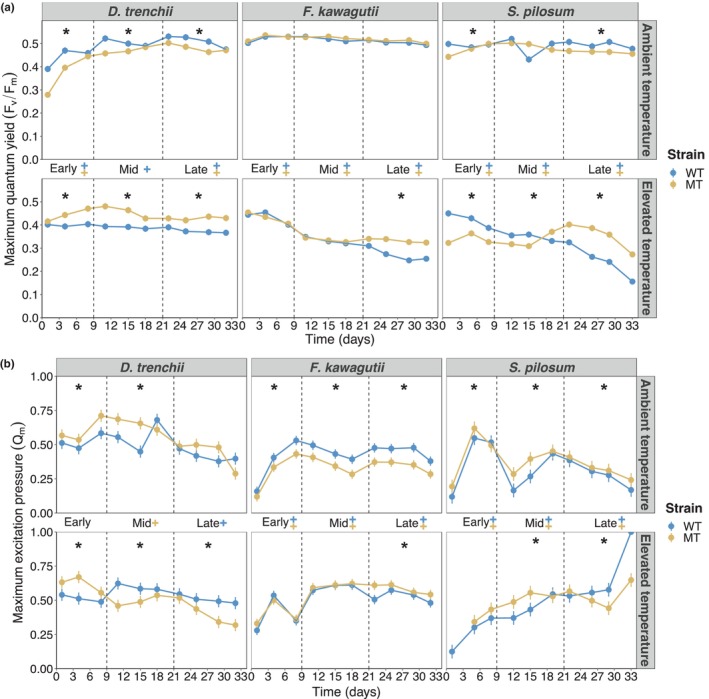
Photosynthetic performances of *Durusdinium trenchii, Fugacium kawagutii* and *Symbiodinium pilosum* cultures. Maximum quantum yield (a) and maximum excitation pressure (b) of photosystem II of mutagen‐treated (MT) and wild‐type (WT) strains at ambient (27°C) and elevated (31°C for *D. trenchii*, 35°C for *F. kawagutii* and *S. pilosum*) temperatures. Significant differences (*p* < 0.01) are indicated for each experimental phase (as defined in Section 2, a). *, differences between both strains at the indicated temperature; +, differences between temperatures for the WT strain; +, differences between temperatures for the MT strain. Error bars represent 95% confidence intervals. *n* = 5 for each strain at each timepoint.

Thermal exposure had a significant negative effect on the *F*_v_/*F*_m_ of both strains (Figure 2a, Table 3). The *F*_v_/*F*_m_ of the WT and MT strains was 43 and 19% lower, respectively, at 31°C than at 27°C in the late experimental phase (*p* < 0.001). At 31°C, the WT strain displayed a *F*_v_/*F*_m_ that was 16%–24% lower than its MT counterpart across all experimental phases (*p* < 0.001). This was accompanied by a higher *Q*_m_ for the WT strain in the latter half of the RTE at 31°C, reflecting greater photoinhibition of the WT lineage under thermal stress (Figure 2b). In contrast, at 27°C the WT strain displayed a higher *F*_v_/*F*_m_ and lower *Q*_m_ than its MT counterpart (*p* < 0.001), showing an improved photosynthetic efficiency.

**FIGURE 3:**
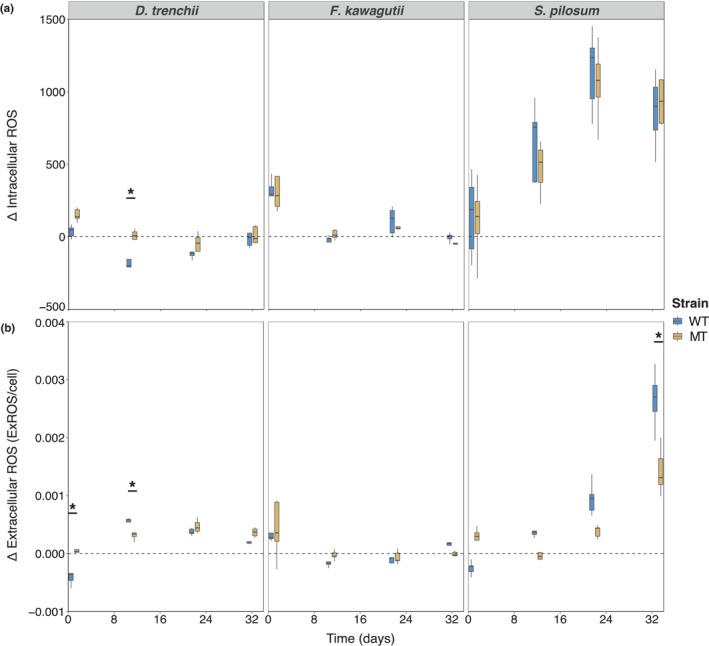
Oxidative stress measurements of *Durusdinium trenchii, Fugacium kawagutii* and *Symbiodinium pilosum* cultures. Differences in intracellular (a) and extracellular (b) reactive oxygen species (ROS) levels between elevated (31°C for *D. trenchii*, 35°C for *F. kawagutii* and *S. pilosum*) and ambient (27°C) temperatures of mutagen‐treated (MT) and wild‐type (WT) strains. Intracellular ROS measurements were obtained from the median fluorescent intensities of Symbiodiniaceae cells stained with CellROX Green. Extracellular ROS values were obtained from the absorbance of cells stained with CellROX Orange and normalized to the cell density measured from the same cultures. Significant differences between strains are only indicated (*p < 0.01). *n* = 5 for each strain at each timepoint.

No differences in intracellular and extracellular ROS levels were recorded between strains by the end of the RTE (Figure 3; Figure S4). Whilst virtually no intracellular ROS was detected, some accumulation of extracellular ROS was measured. However, levels of extracellular ROS were low throughout the RTE and remained stable following day 11, suggesting that both strains may have experienced minimal oxidative stress.

### 3.2 Experimental evolution resulted in improved photochemical efficiency of the MT strain over the WT strain of *F. kawagutii* at 35°C

The elevated temperature had no measurable negative impact on the growth of both *F. kawagutii* strains (Figure 1) and the lowest effect on cell density yields out all three genera (Table 3). Although no differences in growth rates were observed between temperature treatments (Figure 1b), the WT strain displayed 11%–21% higher cell densities at 35 than at 27°C across the whole experiment (*p* < 0.005). The MT strain displayed 26% higher cell densities compared to the WT strain in the late experimental phase (*p* < 0.001; Figure 1a). Whilst the WT strain grew slightly more than its MT counterpart during the mid‐experimental phase (*p* = 0.006) at elevated temperature, after day 25 the cell densities of the WT strain decreased by 14%, compared to the MT strain which grew significantly more by a further 9% (*p* < 0.001). The WT and MT lineages displayed comparable growth rates and cell densities at ambient temperature.

Some degree of thermal stress was experienced by both *F. kawagutii* strains as indicated by a lower *F*_v_/*F*_m_ and higher *Q*_m_ across the experiment at 35°C compared with 27°C (*p* < 0.001; Table 3; Figure 2). At 35°C, both strains displayed a 27% decrease in their *F*_v_/*F*_m_ by day 18. During the late experimental phase, the MT strain maintained a significantly higher *F*_v_/*F*_m_ than the WT strain, which experienced a further 22% decrease in *F*_v_/*F*_m_ (*p* < 0.001). This trend was not reflected in the *Q*_m_ measurements, which remained stable during the latter half of the RTE and even decreased significantly more for the WT strain in the late experimental phase, suggesting lower photoinhibition (*p* < 0.001; Figure 2b). At ambient temperature, both strains displayed a comparable *F*_v_/*F*_m_, though a lower *Q*_m_ was measured for the MT strain throughout the RTE.

No signs of oxidative stress were observed in either strain, which displayed stable low levels of intracellular or extracellular ROS over the course of the RTE (Figure 3). The near‐negligible ROS levels measured suggest that both strains may have experienced little to no oxidative stress at 35°C. Interestingly, both strains displayed slightly higher levels of extracellular ROS on day 1 compared to the subsequent timepoints, at both temperatures (Figure S5). This suggests that handling of the cultures during inoculation may initially induce some degree of stress.

### 3.3 Experimental evolution improved growth, photochemical efficiency and reduced ROS accumulation of the MT *S. pilosum* strain over its WT counterpart at 35°C

Exposure to 35°C had a substantial negative impact on the growth of both *S. pilosum* strains (Table 3), with growth rates found to be significantly lower at 35°C than at 27°C (*p* < 0.001; Figure 1b). Despite similar growth rates at 35°C, thermal stress was experienced sooner in the WT strain (Figure 1a); decreasing cell densities were measured after day 15 for the WT strain compared to day 25 for the MT strain. The MT strain also grew 26% more overall than its WT counterpart (Figure 1a). There was no significant difference in growth at ambient temperature.

Exposure to the elevated temperature had a significant negative effect on the photochemical efficiency of both strains (Table 3). Whilst the WT strain displayed a starting *F*_v_/*F*_m_ 28% higher than the MT strain, a three‐fold decrease of the *F*_v_/*F*_m_ was measured for the WT strain over the course of the RTE (Figure 2a). In comparison, the MT strain displayed a stable *F*_v_/*F*_m_ until day 15, which even increased between days 15–18. Despite decreasing in the final week, the MT strain still displayed an overall *F*_v_/*F*_m_ that was 42% higher than the WT lineage in the late experimental phase (*p* < 0.001). Both strains displayed an increasing *Q*_m_ over the course of the RTE, reflecting worsening photoinhibition (Figure 2b). At 27°C, a slightly higher *Q*_m_ and lower *F*_v_/*F*_m_ were recorded for the MT strain during most of the RTE (*p* < 0.001). Nonetheless, the *F*_v_/*F*_m_ of the MT strain remained high around 0.5 and the *Q*_m_ steadily decreased over the late experimental phase, indicating comparably viable photosystems between strains.

Oxidative stress was experienced by both strains at 35°C, with levels of intracellular and extracellular ROS both increasing considerably at the elevated temperature (Figure 3). Intracellular ROS at 35°C increased at the same rate in the WT and MT strains, with no significant differences observed between both. Extracellular ROS levels at 35°C were significantly higher in the WT strain by the end of the RTE (*p* < 0.001), which were 49% higher than those of the MT strain by the end of the RTE (Figure S6), which may be attributed to the comparatively higher levels of cell death and ensuing cell lysis occurring in the WT strain, resulting in the release of more intracellular ROS. Negligible levels of oxidative stress were recorded in both strains at 27°C (Figure S6), with no differences in ROS levels between the WT and MT lineages recorded.

### 3.4 Nutrient uptake differed within and between species

**FIGURE 4:**
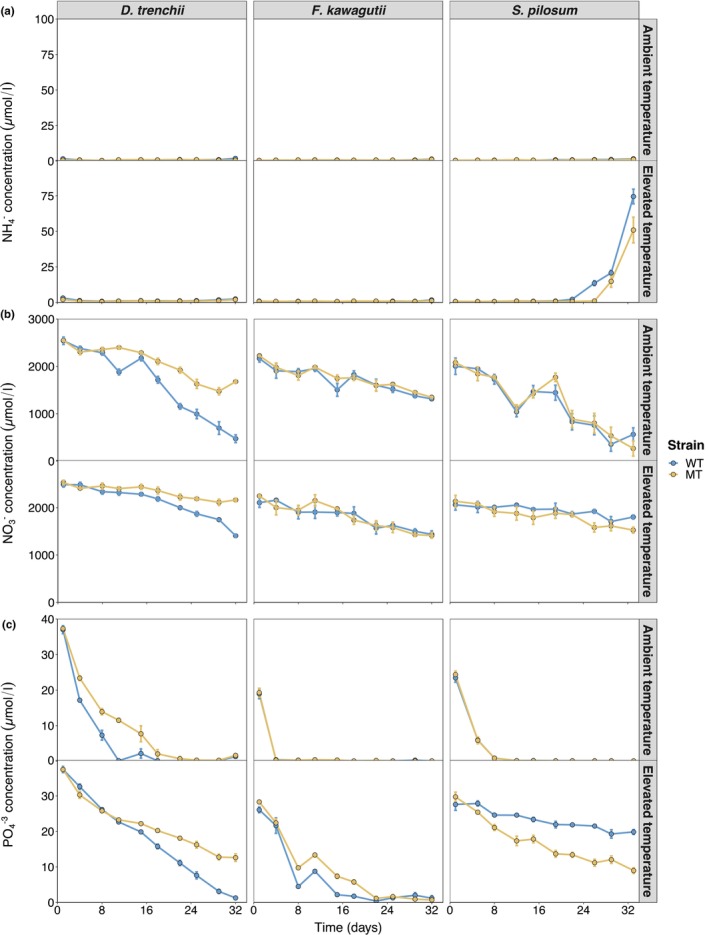
Macronutrient concentrations in the culture media of *Durusdinium trenchii, Fugacium kawagutii* and *Symbiodinium pilosum* cultures. Ammonium (${\mathrm{NH}}_{4}^{+}$; a), nitrate (${\mathrm{NO}}_{3}^{-}$; b) and phosphate (${\mathrm{PO}}_{4}^{-3}$; c) concentrations of mutagen‐treated (MT) and wild‐type (WT) culture media at ambient (27°C) and elevated (31°C for *D. trenchii*, 35°C for *F. kawagutii* and *S. pilosum*) temperatures. Culture media were filtered (0.22 μm) before analysis to remove Symbiodiniaceae cells. Error bars represent 1 standard error. *n* = 5 for each timepoint.

Ammonium concentrations measured in the *D. trenchii*, *F. kawagutii* and *S. pilosum* cultures were negligible (0.4–1.7 μM) for the duration of the RTE (Figure 4a). The ammonium present in the IMK culture medium (63 μM; see Table S1 for macronutrient concentrations measured in a fresh IMK preparation) appeared to have been assimilated within the 24 h that elapsed between inoculation and the first sampling timepoint. Substantial ammonium levels were only recorded in the *S. pilosum* WT and MT cultures at 35°C, which displayed an increase in ammonium after days 22 and 26, reaching 74.5 and 51 μM at the end of the RTE. This increase in extracellular ammonium is most likely explained by cell lysis occurring in these cultures.

**FIGURE 5:**
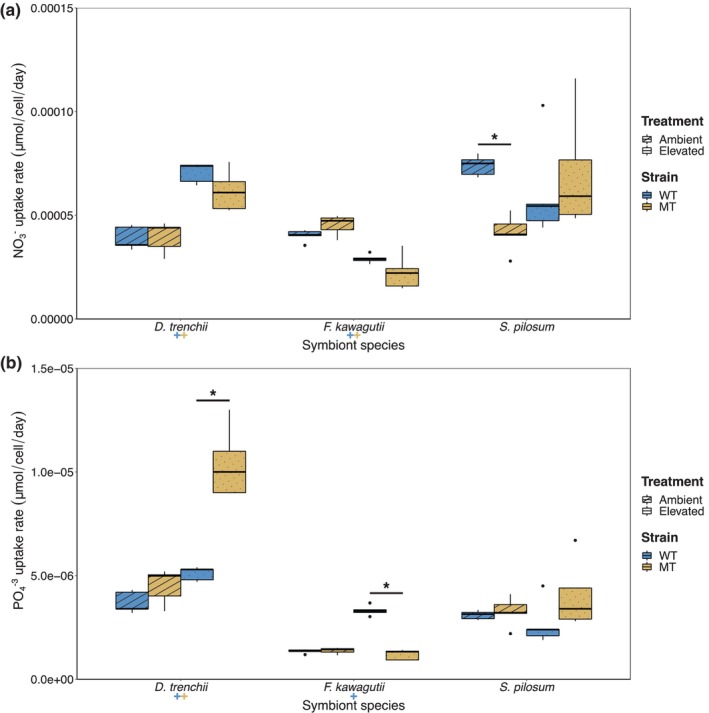
Nitrate and phosphate uptake rates of *Durusdinium trenchii, Fugacium kawagutii* and *Symbiodinium pilosum* cultures. Nitrate (${\mathrm{NO}}_{3}^{-}$; a) and phosphate (${\mathrm{PO}}_{4}^{-3}$; b) uptake rates of mutagen‐treated (MT) and wild‐type (WT) cultures at ambient (27°C) and elevated (31°C for *D. trenchii*, 35°C for *F. kawagutii* and *S. pilosum*) temperatures. *, significant differences (*p* < 0.01) between both strains at the indicated temperature; +, differences between temperatures for the WT strain; +, differences between temperatures for the MT strain. *n* = 5 for each timepoint.

Despite comparable cell densities between *S. pilosum* and *F*. *kawagutii* at 27°C, nitrate concentrations decreased considerably more in the *S. pilosum* cultures than in the *F. kawagutii* cultures (267–563 vs. 1314–1347 μM by the end of the RTE, Figure 4b). Nitrate uptake rates were significantly affected by temperature in *D. trenchii* and *F. kawagutii* (Table 3), though contrasting responses were observed. Higher and lower nitrate uptake rates were measured for WT and MT cultures from *D. trenchii* and *F. kawagutii*, respectively, at elevated temperature (*p* < 0.002). No differences in nitrate uptake rates were measured between respective *D. trenchii* and *F. kawagutii* strains at either temperature, though nitrate assimilation rates were lower in the *S. pilosum* MT cultures at 27°C than in the respective WT cultures (*p* < 0.001; Figure 5a).

At 27°C, phosphate became rapidly depleted in the *F. kawagutii* and *S. pilosum* cultures, within the first week of growth, and within 18 days in the *D. trenchii* cultures (Figure 4c). Thermal exposure had a significant effect on phosphate uptake rates in *D. trenchii* and *F. kawagutii* (Table 3), with WT cultures from both species displaying greater phosphate requirements at 31/35°C (*p* < 0.003, Figure 5b). Furthermore, MT cultures from *D. trenchii* and *F. kawagutii* displayed higher and lower, respectively, phosphate uptake rates compared to their WT counterparts (*p* < 0.001).

## 4 DISCUSSION

Comparisons of the physiological performances of MT and WT strains from *D. trenchii*, *F. kawagutii* and *S. pilosum* at ambient (27°C) and elevated temperatures (31/35°C) permitted the detection of signatures of stable adaptive change to thermal stress following chemical mutagenesis and thermal selection. Despite originating from highly divergent taxa (González‐Pech et al., 2021) and differing in their thermotolerance strategies, all three MT strains displayed an improvement in their photochemical efficiency under thermal stress. However, trade‐offs in the MT strains were identified as a result of experimental evolution.

### 4.1 Increased photosynthetic efficiency under thermal stress comes with growth trade‐offs in the *D. trenchii* heat‐evolved strain

**FIGURE 6:**
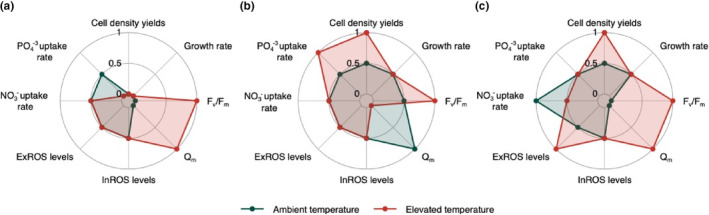
Summary of differences in physiological performances between mutagen‐treated (MT) and wild‐type (WT) strains at ambient and elevated temperatures (a = *D. trenchii*, b = *F. kawagutii*, c = *S. pilosum*). A score of 0 represents a decrease in trait performance by the MT strain relative to its WT counterpart, a score of 0.5 denotes the absence of change in trait performance between both strains, and a score of 1 represents an increase in trait performance by the MT strain. For time series data (i.e. cell density yields, *F*_v_/*F*_m_ and *Q*_m_) the assessment is based on differences in the late experimental phase.

At the elevated temperature, the higher *F*_v_/*F*_m_ and lower *Q*_m_ of the *D. trenchii* MT strain relative to its WT counterpart suggests that experimental evolution has led to an improvement in the photochemical efficiency of this species under thermal stress (Figure 6). However, this increased thermal tolerance was accompanied by a trade‐off against growth in that *D. trenchii* MT strain has a lower growth rate relative to the WT strain at both temperature treatments. The lower *F*_v_/*F*_m_ experienced by both strains at 31°C compared to 27°C indicates that heat had a negative impact on the photosynthetic efficiency of this species, implying it suffered some degree of thermal stress. However, the low ROS levels measured at 31°C suggest that minimal oxidative stress was experienced in both strains, either due to a lower amount of ROS having been produced and/or to a high ROS‐mitigating capacity in *D. trenchii*.

In a previous experimental evolution attempt, the same *D. trenchii* strain (SCF086.01) displayed reduced growth and photosynthetic performance compared to its WT counterpart after thermal selection only (1 year/25–26 generations at 30°C) (Chakravarti & van Oppen, 2018). Our second attempt to evolve *D. trenchii*, via EMS exposure and thermal selection at 31°C, resulted in a slightly improved in vitro thermal tolerance (higher photochemical efficiency at 31°C during the RTE).

*D. trenchii* is often reported as being one of the most thermally tolerant symbionts found in scleractinian corals (Berkelmans & van Oppen, 2006; Mieog et al., 2009; Silverstein et al., 2017). A potential explanation for the difficulty to increase the thermal tolerance of *D. trenchii* is that its adaptive capacity to warmer temperatures is limited. It is possible the whole genome duplication that has occurred in *D. trenchii* (Dougan et al., 2022) may be restricting the ability of experimental evolution to induce adaptation, as thermal selection may need to act on both copies of genes underpinning thermal tolerance to have a measurable phenotypic effect.

Two limitations of this study are that only one MT strain was tested and that we selected the fastest‐growing lineages for the RTEs. Given that mutations occur randomly in each cell division and those induced by the mutagen are also random, the outcome of experimental evolution is expected to differ among replicates, which was confirmed for heat‐evolved strains of *Cladocopium proliferum* (Buerger et al., 2020).

Most experimental evolution studies on marine microalgae have employed exposure to stable, elevated temperatures (Chan et al., 2021), but alternative experimental evolution approaches may be required for *D. trenchii*. This may include exposure to fluctuating rather than stable elevated temperatures, which has yielded improved adaptation to severe warming in the marine diatom *T. pseudonana* (Schaum et al., 2018), and/or to repeated cycles of mutagenesis and thermal selection, which has resulted in a 2°C increase in thermal tolerance in the cyanobacteria *Synechocystis* sp. (Tillich et al., 2012).

### 4.2 Chemical mutagenesis followed by thermal selection improved the in vitro thermal tolerance of *S. pilosum* and *F. kawagutii*

Compared to their respective WT counterparts, the *F. kawagutii* and *S. pilosum* MT strains maintained higher cell densities and photochemical efficiency under thermal stress (Figure 6). Whilst the *F. kawagutii* strains displayed no signs of oxidative stress, both *S. pilosum* strains experienced high levels of oxidative stress at 35°C, although the heat‐evolved lineage accumulated less extracellular ROS. No trade‐off between thermal tolerance and growth was recorded in the heat‐evolved strains under ambient versus elevated conditions. A slightly decreased photosynthetic performance was recorded for the heat‐evolved *F. kawagutii* strain at 35°C and *S. pilosum* strain at 27°C. Our results suggest that chemical mutagenesis followed by thermal selection successfully increased the in vitro thermal tolerance of the *F. kawagutii* and *S. pilosum* MT strains, providing two new heat‐evolved strains with increased thermal tolerance.

The genus *Symbiodinium* displays significant inter‐species variability in in vitro thermal tolerance (Díaz‐Almeyda et al., 2017; Krueger et al., 2014). Our findings support previous observations that *S. pilosum* is a thermally tolerant species in this genus (Díaz‐Almeyda et al., 2017), with little to no decrease in *F*_v_/*F*_m_ of WT *S. pilosum* measured at 31–34°C (Figures S2 and S3). In a previous study, cultures from the genus *Symbiodinium* were found to display alternative photosynthetic electron pathways compared to members from the genera *Breviolum*, *Cladocopium*, *Durusdinium* and *Fugacium* (Reynolds et al., 2008). Constitutive cyclic electron transport, providing an alternative ATP synthesis pathway, and chlororespiration, mitigating ROS formation, in *Symbiodinium* are mechanisms that may help improve the survival of members from this genus under thermal perturbation of the photosystem II.

The higher cell densities of the *F. kawagutii* WT strain at 35 than at 27°C and lack of oxidative stress indicate a high innate thermal tolerance for this species, corroborating previous studies that have demonstrated a high thermal tolerance of *F. kawagutii* (Chakravarti & van Oppen, 2018; Díaz‐Almeyda et al., 2011; Krueger et al., 2014). In a genomic analysis of a *F. kawagutii* strain, Lin et al. (2015) identified the presence of expanded gene families encoding heat shock proteins and DNA repair proteins, as well as a large set of antioxidant genes, which may explain the high thermal tolerance of this species. In a separate study, a proportionally greater expression of antioxidant genes was also found in another *F. kawagutii* strain, compared to strains from the genera *Durusdinium*, *Cladocopium* and *Breviolum* at 33°C (Krueger et al., 2014). The low intracellular and extracellular ROS levels in both *F. kawagutii* strains at 35°C, despite the reduction in photosynthetic efficiency, support previous observations that the high thermal tolerance of *F. kawagutii* may be attributed to this species' capacity to mitigate oxidative stress.

### 4.3 Rapid depletion of ammonium and phosphate in the culture medium may constrain Symbiodiniaceae growth in vitro

In the context of the oligotrophic conditions characteristic of tropical coral reefs, *in hospite* Symbiodiniaceae growth is limited by the availability of dissolved inorganic nitrogen (mainly ammonium and nitrate), phosphate or inorganic carbon (CO_2_; Morris et al., 2019). Amongst the different environmental sources of inorganic nitrogen assimilated by Symbiodiniaceae, ammonium is the preferred source of nitrogen over nitrate (Grover et al., 2002, 2003; Pernice et al., 2012), which was reflected by the rapid depletion of ammonium across all cultures in our study. The negligible ammonium and phosphate levels in the media observed for all three species at ambient temperature suggest that growth may have been primarily limited by these two nutrients, although the high nitrate levels measured across all three species after a month of growth at ambient temperature indicate that the microalgae were not nitrogen‐limited. An enriched f/2 culture medium containing twice the starting phosphate, silicate, microelement and vitamin concentrations prevented the limitation of growth by nutrient depletion in a marine diatom population (Orefice et al., 2019). As such, increasing the starting concentrations of macronutrients (ammonium and phosphate), microelements and vitamins in growth media used for Symbiodiniaceae cultivation may be a potential avenue for optimizing growth rates.

### 4.4 Thermal stress and experimental evolution affect symbiont nutrient acquisition dynamics

Thermal stress had a significant effect on the nitrate uptake rates of *D. trenchii* and *F. kawagutii* cultures, though contrasting responses were observed between both species. Our results support previous observations that nitrate assimilation increases in *D. trenchii* under thermal stress (Baker et al., 2013; McIlroy et al., 2020). Unexpectedly, the *F. kawagutii* strains displayed lower nitrate uptake rates at elevated than at ambient temperatures, whilst growing more. Members from the genus *Fugacium* have been previously described as velocity‐adapted with regard to their nitrate consumption, ensuring that assimilated nitrate is used for growth rather than storage (Wong et al., 2021). This would explain the high growth rates displayed by both *F. kawagutii* strains, relative to other taxa in our study. Nitrate uptake rates were comparable between temperatures in *S. pilosum* cultures, despite heat displaying a high negative effect on other physiological traits. The reduced nitrate requirement of the MT cultures over their WT counterpart at ambient temperature indicates that experimental evolution may have altered some nutrition resource usage and allocation processes of the heat‐evolved lineage.

Under thermal stress, *in hospite* Symbiodiniaceae have been reported to increase their phosphate consumption (Godinot et al., 2011). This observation is corroborated here by the increased phosphate uptake rates recorded in the *D. trenchii* and *F. kawagutii* WT cultures under thermal stress, although temperature once again had no effect on *S. pilosum* cultures. Phosphate starvation in corals has been found to lead to an altered composition of photosymbiont thylakoid membranes for light/thermal damage, thus lowering the bleaching threshold of the coral holobiont (Wiedenmann et al., 2013). Hence, in vitro phosphate requirements of Symbiodiniaceae cultures under thermal stress could be an important indicator to identify strains that will translate their in vitro thermal tolerance to an improved bleaching resilience *in hospite*. At elevated temperature, the lower phosphate uptake rate measured in the *F. kawagutii* MT cultures relative to the WT cultures can be considered as additional evidence that experimental evolution improved the capacity of the heat‐evolved lineage to withstand thermal stress. Conversely, the increased phosphate requirement of the *D. trenchii* MT cultures represents an additional trade‐off, although this apparently did not result in a reduced photosynthetic efficiency under thermal stress as could have been expected.

### 4.5 Acceptable trade‐offs to improve coral thermal tolerance

Trade‐offs are expected to occur following experimental evolution, given they are intricately linked to life‐history evolution (Stearns, 1989). Here, we report trade‐offs in traits among MT lineages that range in magnitude across growth, photochemical efficiency and nutrient usage. EMS mutagenesis is known to induce point mutations randomly across the genome of the target organism (Lethin et al., 2020; McCallum et al., 2000). The stochastic nature of this process means that some mutations are likely to have been neutral or deleterious. The absence of mortality recorded across the MT lineages following EMS exposure (Table 1) suggests that 100 mM was insufficient to cause toxic side effects, as was observed by Doan and Obbard (2012). The high mortality across MT lineages recorded following 4 years of thermal selection suggests that exposure to elevated temperatures led to the elimination of genotypes that lacked sufficient heat adaptation.

From the perspective of assisted evolution efforts to improve coral thermal tolerance, some of the observed costs incurred to the fitness of the heat‐evolved lineages could be considered as ‘acceptable trade‐offs’. For instance, any negative impact of a reduction in growth in vitro may be limited *in hospite*, considering that the coral host tends to restrict the proliferation of its symbionts through nutritional regulation anyway (Cui et al., 2022). Improvements in photochemical efficiency under thermal stress, observed across all MT strains in our study, could provide benefits to coral thermal tolerance that outweigh the costs of reduced growth since the deterioration of the Symbiodiniaceae photosynthetic apparatus is inducive to coral bleaching (Weis, 2008). However, increases in nutrient uptake, such as the increased phosphate requirement of the MT *D. trenchii* lineage, could negatively impact the nutritional balance between the host and algal symbiont (Godinot et al., 2011). More broadly, determining which in vitro phenotypes, if any, confer an improved thermal tolerance *in hospite* would be highly valuable to rapidly identify heat‐evolved strains for coral reef restoration efforts (Buerger et al., 2023).

### 4.6 *S. pilosum* and *F. kawagutii* as novel symbionts for coral reef restoration

Representatives from the genera *Symbiodinium* and *Fugacium* are not commonly found as dominant symbionts in Indo‐Pacific corals (Baker, 2003). Nonetheless, background symbionts (less than ~1% abundance) are often present in corals and some reports suggest that these rare symbionts may contribute to the environmental resilience of the coral holobiont (Boulotte et al., 2016; Qin et al., 2019; Ziegler et al., 2018).

*Symbiodinium* strains are increasingly recorded in corals (Camp et al., 2020; Lajeunesse et al., 2009; Stat & Gates, 2008) and have been found to play an important role in the survival of some *Acropora* larvae, with higher proportional abundances of *S. tridacnidorum* and *Symbiodinium* sp. reported to reduce mortality in *A. tenuis* and *A. yongei* juveniles, respectively (Quigley et al., 2016; Suzuki et al., 2013). These reports indicate that coral‐*Symbiodinium* associations may be more widespread than previously anticipated in the Indo‐Pacific. A recent study also showed an increased capacity of *Acropora tenuis* larvae to acquire *Fugacium* symbionts at warmer temperatures (Matsuda et al., 2022), suggesting these rare symbionts may increase in dominance in warming oceans.

Future work should explore the adult host range of these thermo‐tolerant symbionts, particularly among Acroporidae where the uptake of *Symbiodinium* strains occurs and appears beneficial to the host thermal tolerance. This could be achieved through chemical bleaching and reinoculation of adult coral fragments, an approach that has recently proven successful in enabling the uptake of heterologous symbionts (Scharfenstein et al., 2022). If these rare symbionts become stable members of the coral holobiont, they may be promising candidates for enhancing the thermal tolerance of coral stock used for restoration.

Algal symbiont manipulation efforts should examine the effects of combining experimentally evolved symbionts with native symbionts, an approach that may be used to maintain or increase the Symbiodiniaceae diversity of corals while incorporating enhanced strains. An elevated symbiont diversity can provide redundant or complementary symbiotic functions, a key factor to increase the stability of the coral‐Symbiodiniaceae symbiosis in the face of increasing environmental disturbances (Fabina et al., 2013). Given the high degree of symbiont specificity in certain coral species (e.g. C15 in *Porites* spp.; Camp et al., 2020), a key element to achieve this is to expand the taxonomic diversity of cultured and heat‐evolved Symbiodiniaceae. Finally, incorporating freshly isolated symbionts in in vitro thermal tolerance assessments would be highly valuable to better compare gains in thermal tolerance of heat‐evolved Symbiodiniaceae to the environmental reality.

## 5 CONCLUSIONS

This study expands the suite of heat‐evolved Symbiodiniaceae with augmented in vitro thermal tolerance, adding MT strains of the species *D. trenchii*, *F. kawagutii* and *S. pilosum* that displayed improved stable adaptive responses to thermal stress following chemical mutagenesis and thermal selection. Further research into the optimization of chemical mutagenesis and thermal selection may reveal whether this can provide a faster experimental evolution approach to increase microalgal thermal tolerance compared to thermal selection alone. The rapid depletion of ammonium and phosphate measured across all cultures suggests that increasing starting concentrations of these macronutrients in microalgal culture media may be a promising avenue to optimize Symbiodiniaceae growth rates in vitro. Given the growing recognition of the role of nutrient cycling between the coral host and the Symbiodiniaceae in coral bleaching, incorporating assessments of Symbiodiniaceae macronutrient usage in in vitro studies may help better identify symbionts that will confer an improved bleaching resilience to their host. The high in vitro thermal tolerance reported here in the *S. pilosum* and *F. kawagutii* strains make these strong candidates to develop novel coral‐Symbiodiniaceae symbioses and test these for improved bleaching tolerance.

## CONFLICT OF INTEREST STATEMENT

The authors declare no conflict of interest.

## Supporting information


Data S1

## Data Availability

The dataset associated with this study has been archived at the Australian Institute of Marine Science's Research Data Platform. The dataset can be accessed at: https://apps.aims.gov.au/metadata/view/a86b9bca‐1982‐4669‐ab81‐a1c6fb8c583e. Code associated with this study can be found at: https://github.com/hscharfenstein/Chemical‐mutagenesis‐and‐thermal‐selection‐of‐Symbiodiniaceae.

## References

[eva13586-bib-0001] Baker, A. C. (2003). Flexibility and specificity in coral‐algal symbiosis: Diversity, ecology, and biogeography of *Symbiodinium* . Annual Review of Ecology, Evolution, and Systematics, 34, 661–689. 10.1146/annurev.ecolsys.34.011802.132417

[eva13586-bib-0002] Baker, D. M. , Andras, J. P. , Jordán‐Garza, A. G. , & Fogel, M. L. (2013). Nitrate competition in a coral symbiosis varies with temperature among *Symbiodinium* clades. The ISME Journal, 7(6), 1248–1251. 10.1038/ismej.2013.12 23407311 PMC3660672

[eva13586-bib-0003] Berkelmans, R. , & Van Oppen, M. J. H. (2006). The role of zooxanthellae in the thermal tolerance of corals: A “nugget of hope” for coral reefs in an era of climate change. Proceedings of the Royal Society B: Biological Sciences, 273(1599), 2305–2312. 10.1098/rspb.2006.3567 PMC1636081 16928632

[eva13586-bib-0004] Boulotte, N. M. , Dalton, S. J. , Carroll, A. G. , Harrison, P. L. , Putnam, H. M. , Peplow, L. M. , & Van Oppen, M. J. H. (2016). Exploring the *Symbiodinium* rare biosphere provides evidence for symbiont switching in reef‐building corals. The ISME Journal, 10(11), 2693–2701. 10.1038/ismej.2016.54 27093048 PMC5113844

[eva13586-bib-0005] Brown, B. E. (1997). Coral bleaching: Causes and consequences. Coral Reefs, 16, 129–138.

[eva13586-bib-0006] Buerger, P. , Alvarez‐Roa, C. , Coppin, C. W. , Pearce, S. L. , Chakravarti, L. J. , Oakeshott, J. G. , Edwards, O. R. , & van Oppen, M. J. H. (2020). Heat‐evolved microalgal symbionts increase coral bleaching tolerance. Science Advances, 6(20), 1–9. 10.1126/sciadv.aba2489 PMC7220355 32426508

[eva13586-bib-0007] Buerger, P. , Buler, M. , Yeap, H. L. , Edwards, O. R. , van Oppen, M. J. H. , Oakeshott, J. G. , & Court, L. (2023). Flow cytometry‐based biomarker assay for *in vitro* identification of microalgal symbionts conferring heat tolerance on corals. Frontiers in Marine Science, 10, 1094792. 10.3389/fmars.2023.1094792

[eva13586-bib-0008] Butler, C. C. , Turnham, K. E. , Lewis, A. M. , Nitschke, M. R. , Warner, M. E. , Kemp, D. W. , Hoegh‐Guldberg, O. , Fitt, W. K. , van Oppen, M. J. H. , & LaJeunesse, T. C. (2023). Formal recognition of host‐generalist species of dinoflagellate (*Cladocopium*, Symbiodiniaceae) mutualistic with indo‐Pacific reef corals. Journal of Phycology, 59, 698–711. 10.1111/JPY.13340 37126002

[eva13586-bib-0009] Camp, E. F. , Suggett, D. J. , Pogoreutz, C. , Nitschke, M. R. , Houlbreque, F. , Hume, B. C. C. , Gardner, S. G. , Zampighi, M. , Rodolfo‐Metalpa, R. , & Voolstra, C. R. (2020). Corals exhibit distinct patterns of microbial reorganisation to thrive in an extreme inshore environment. Coral Reefs, 39(3), 701–716. 10.1007/s00338-019-01889-3

[eva13586-bib-0010] Cantin, N. E. , Van Oppen, M. J. H. , Willis, B. L. , Mieog, J. C. , & Negri, A. P. (2009). Juvenile corals can acquire more carbon from high‐performance algal symbionts. Coral Reefs, 28(2), 405–414. 10.1007/s00338-009-0478-8

[eva13586-bib-0011] Chakravarti, L. J. , Beltran, V. H. , & van Oppen, M. J. H. (2017). Rapid thermal adaptation in photosymbionts of reef‐building corals. Global Change Biology, 23(11), 4675–4688. 10.1111/gcb.13702 28447372

[eva13586-bib-0012] Chakravarti, L. J. , & van Oppen, M. J. H. (2018). Experimental evolution in coral photosymbionts as a tool to increase thermal tolerance. Frontiers in Marine Science, 5, 227. 10.3389/fmars.2018.00227

[eva13586-bib-0013] Chan, W. Y. , Oakeshott, J. G. , Buerger, P. , Edwards, O. R. , & van Oppen, M. J. H. (2021). Adaptive responses of free‐living and symbiotic microalgae to simulated future ocean conditions. Global Change Biology, 27(9), 1737–1754. 10.1111/gcb.15546 33547698

[eva13586-bib-0014] Chou, H. H. , Su, H. Y. , Di Song, X. , Chow, T. J. , Chen, C. Y. , Chang, J. S. , & Lee, T. M. (2019). Isolation and characterization of *Chlorella* sp. mutants with enhanced thermo‐ and CO_2_ tolerances for CO_2_ sequestration and utilization of flue gases. Biotechnology for Biofuels, 12(1), 251. 10.1186/s13068-019-1590-9 31641373 PMC6800494

[eva13586-bib-0015] Coffroth, M. A. , Lewis, C. F. , Santos, S. R. , & Weaver, J. L. (2006). Environmental populations of symbiotic dinoflagellates in the genus *Symbiodinium* can initiate symbioses with reef cnidarians. Current Biology, 16(23), 985–987. 10.1016/j.cub.2006.10.049 17141602

[eva13586-bib-0016] Cui, G. , Liew, Y. J. , Konciute, M. K. , Zhan, Y. , Hung, S. H. , Thistle, J. , Gastoldi, L. , Schmidt‐Roach, S. , Dekker, J. , & Aranda, M. (2022). Nutritional control regulates symbiont proliferation and life history in coral‐dinoflagellate symbiosis. BMC Biology, 20(1), 103. 10.1186/s12915-022-01306-2 35549698 PMC9102920

[eva13586-bib-0017] Davies, P. S. (1984). The role of zooxanthellae in the nutritional energy requirements of *Pocillopora eydouxi* . Coral Reefs, 2(4), 181–186. 10.1007/BF00263571

[eva13586-bib-0018] Davy, S. K. , Allemand, D. , & Weis, V. M. (2012). Cell biology of cnidarian‐dinoflagellate symbiosis. Microbiology and Molecular Biology Reviews, 76(2), 229–261. 10.1128/mmbr.05014-11 22688813 PMC3372257

[eva13586-bib-0019] Díaz‐Almeyda, E. , Thomé, P. E. , El Hafidi, M. , & Iglesias‐Prieto, R. (2011). Differential stability of photosynthetic membranes and fatty acid composition at elevated temperature in *Symbiodinium* . Coral Reefs, 30(1), 217–225. 10.1007/s00338-010-0691-5

[eva13586-bib-0020] Díaz‐Almeyda, E. M. , Prada, C. , Ohdera, A. H. , Moran, H. , Civitello, D. J. , Iglesias‐Prieto, R. , Carlo, T. A. , Lajeunesse, T. C. , & Medina, M. (2017). Intraspecific and interspecific variation in thermotolerance and photoacclimation in *Symbiodinium* dinoflagellates. Proceedings of the Royal Society B: Biological Sciences, 284(1868), 20171767. 10.1098/rspb.2017.1767 PMC5740277 29212723

[eva13586-bib-0021] Doan, T. T. Y. , & Obbard, J. P. (2012). Enhanced intracellular lipid in *Nannochloropsis* sp. via random mutagenesis and flow cytometric cell sorting. Algal Research, 1(1), 17–21. 10.1016/j.algal.2012.03.001

[eva13586-bib-0022] Dougan, K. E. , Bellantuono, A. J. , Kahlke, T. , Abbriano, R. M. , Chen, Y. , Shah, S. , Granados‐Cifuentes, C. , van Oppen, M. J. H. , Bhattacharya, D. , Suggett, D. J. , Chan, C. X. , & Rodriguez‐Lanetty, M. (2022). Whole‐genome duplication in an algal symbiont serendipitously confers thermal tolerance to corals. *BioRxiv*, 1–19. 10.1101/2022.04.10.487810 PMC11259175 39028812

[eva13586-bib-0023] Fabina, N. S. , Putnam, H. M. , Franklin, E. C. , Stat, M. , & Gates, R. D. (2012). Transmission mode predicts specificity and interaction patterns in coral‐*Symbiodinium* networks. PLoS One, 7(9), e44970. 10.1371/journal.pone.0044970 23028711 PMC3445617

[eva13586-bib-0024] Fabina, N. S. , Putnam, H. M. , Franklin, E. C. , Stat, M. , & Gates, R. D. (2013). Symbiotic specificity, association patterns, and function determine community responses to global changes: Defining critical research areas for coral‐*Symbiodinium* symbioses. Global Change Biology, 19(11), 3306–3316. 10.1111/gcb.12320 23847174

[eva13586-bib-0025] Falkowski, P. G. , Dubinsky, Z. , Muscatine, L. , & Porter, J. W. (1984). Light and the bioenergetics of a symbiotic coral. Bioscience, 34(11), 705–709. 10.2307/1309663

[eva13586-bib-0026] Fisch, J. , Drury, C. , Towle, E. K. , Winter, R. N. , & Miller, M. W. (2019). Physiological and reproductive repercussions of consecutive summer bleaching events of the threatened Caribbean coral *Orbicella faveolata* . Coral Reefs, 38(4), 863–876. 10.1007/s00338-019-01817-5

[eva13586-bib-0027] Fujise, L. , Suggett, D. J. , Stat, M. , Kahlke, T. , Bunce, M. , Gardner, S. G. , Goyen, S. , Woodcock, S. , Ralph, P. J. , Seymour, J. R. , Siboni, N. , & Nitschke, M. R. (2021). Unlocking the phylogenetic diversity, primary habitats, and abundances of free‐living Symbiodiniaceae on a coral reef. Molecular Ecology, 30(1), 343–360. 10.1111/mec.15719 33141992

[eva13586-bib-0028] Godinot, C. , Houlbrèque, F. , Grover, R. , & Ferrier‐Pagès, C. (2011). Coral uptake of inorganic phosphorus and nitrogen negatively affected by simultaneous changes in temperature and pH. PLoS One, 6(9), e25024. 10.1371/journal.pone.0025024 21949839 PMC3174978

[eva13586-bib-0029] González‐Pech, R. A. , Stephens, T. G. , Chen, Y. , Mohamed, A. R. , Cheng, Y. , Shah, S. , Dougan, K. E. , Fortuin, M. D. A. , Lagorce, R. , Burt, D. W. , Bhattacharya, D. , Ragan, M. A. , & Chan, C. X. (2021). Comparison of 15 dinoflagellate genomes reveals extensive sequence and structural divergence in family Symbiodiniaceae and genus Symbiodinium. BMC Biology, 19(1), 1–22. 10.1186/S12915-021-00994-6 33849527 PMC8045281

[eva13586-bib-0030] Grover, R. , Maguer, J.‐F. , Allemand, D. , & Ferrier‐Pagè, C. (2003). Nitrate uptake in the scleractinian coral *Stylophora pistillata* . Limnology and Oceanography, 48(6), 2266–2274. 10.4319/lo.2003.48.6.2266

[eva13586-bib-0031] Grover, R. , Maguer, J.‐F. , Reynaud‐Vaganay, S. , & Ferrier‐Pagè, C. (2002). Uptake of ammonium by the scleractinian coral *Stylophora pistillata*: Effect of feeding, light, and ammonium concentrations. Limnology and Oceanography, 47(3), 782–790. 10.4319/lo.2002.47.3.0782

[eva13586-bib-0032] Hall, B. G. , Acar, H. , Nandipati, A. , & Barlow, M. (2014). Growth rates made easy. Molecular Biology and Evolution, 31(1), 232–238. 10.1093/molbev/mst187 24170494

[eva13586-bib-0033] Hoegh‐Guldberg, O. (1999). Climate change, coral bleaching and the future of the world's coral reefs. Marine and Freshwater Research, 50(8), 839–866. 10.1071/MF99078

[eva13586-bib-0034] Hoffmann, A. A. , & Sgró, C. M. (2011). Climate change and evolutionary adaptation. Nature, 470(7335), 479–485. 10.1038/nature09670 21350480

[eva13586-bib-0035] Howells, E. J. , Beltran, V. H. , Larsen, N. W. , Bay, L. K. , Willis, B. L. , & van Oppen, M. J. H. (2012). Coral thermal tolerance shaped by local adaptation of photosymbionts. Nature Climate Change, 2(2), 116–120. 10.1038/nclimate1330

[eva13586-bib-0036] Huertas, E. I. , Rouco, M. , López‐Rodas, V. , & Costas, E. (2011). Warming will affect phytoplankton differently: Evidence through a mechanistic approach. Proceedings of the Royal Society B: Biological Sciences, 278(1724), 3534–3543. 10.1098/rspb.2011.0160 PMC3189365 21508031

[eva13586-bib-0037] Hughes, T. P. , Kerry, J. T. , Álvarez‐Noriega, M. , Álvarez‐Romero, J. G. , Anderson, K. D. , Baird, A. H. , Babcock, R. C. , Beger, M. , Bellwood, D. R. , Berkelmans, R. , Bridge, T. C. , Butler, I. R. , Byrne, M. , Cantin, N. E. , Comeau, S. , Connolly, S. R. , Cumming, G. S. , Dalton, S. J. , Diaz‐Pulido, G. , … Wilson, S. K. (2017). Global warming and recurrent mass bleaching of corals. Nature, 543(7645), 373–377. 10.1038/nature21707 28300113

[eva13586-bib-0038] Hughes, T. P. , Kerry, J. T. , Baird, A. H. , Connolly, S. R. , Dietzel, A. , Eakin, C. M. , Heron, S. F. , Hoey, A. S. , Hoogenboom, M. O. , Liu, G. , McWilliam, M. J. , Pears, R. J. , Pratchett, M. S. , Skirving, W. J. , Stella, J. S. , & Torda, G. (2018). Global warming transforms coral reef assemblages. Nature, 556(7702), 492–496. 10.1038/s41586-018-0041-2 29670282

[eva13586-bib-0039] Hughes, T. P. , Kerry, J. T. , Connolly, S. R. , Baird, A. H. , Eakin, C. M. , Heron, S. F. , Hoey, A. S. , Hoogenboom, M. O. , Jacobson, M. , Liu, G. , Pratchett, M. S. , Skirving, W. , & Torda, G. (2019). Ecological memory modifies the cumulative impact of recurrent climate extremes. Nature Climate Change, 9(1), 40–43. 10.1038/s41558-018-0351-2

[eva13586-bib-0040] Iglesias‐Prieto, R. , Beltrán, V. H. , LaJeunesse, T. C. , Reyes‐Bonilla, H. , & Thomé, P. E. (2004). Different algal symbionts explain the vertical distribution of dominant reef corals in the eastern Pacific. Proceedings of the Royal Society B: Biological Sciences, 271(1549), 1757–1763. 10.1098/rspb.2004.2757 PMC1691786 15306298

[eva13586-bib-0041] Jones, A. , & Berkelmans, R. (2010). Potential costs of acclimatization to a warmer climate: Growth of a reef coral with heat tolerant vs. sensitive symbiont types. PLoS One, 5(5), e10437. 10.1371/journal.pone.0010437 20454653 PMC2862701

[eva13586-bib-0042] Krueger, T. , Becker, S. , Pontasch, S. , Dove, S. , Hoegh‐Guldberg, O. , Leggat, W. , Fisher, P. L. , & Davy, S. K. (2014). Antioxidant plasticity and thermal sensitivity in four types of *Symbiodinium* sp. Journal of Phycology, 50(6), 1035–1047. 10.1111/jpy.12232 26988785

[eva13586-bib-0043] LaJeunesse, T. C. (2002). Diversity and community structure of symbiotic dinoflagellates from Caribbean coral reefs. Marine Biology, 141(2), 387–400. 10.1007/s00227-002-0829-2

[eva13586-bib-0044] Lajeunesse, T. C. , Loh, W. , & Trench, R. K. (2009). Do introduced endosymbiotic dinoflagellates “take” to new hosts? Biological Invasions, 11(4), 995–1003. 10.1007/s10530-008-9311-5

[eva13586-bib-0045] LaJeunesse, T. C. , Parkinson, J. E. , Gabrielson, P. W. , Jeong, H. J. , Reimer, J. D. , Voolstra, C. R. , & Santos, S. R. (2018). Systematic revision of Symbiodiniaceae highlights the antiquity and diversity of coral endosymbionts. Current Biology, 1–11, 2570–2580.e6. 10.1016/j.cub.2018.07.008 30100341

[eva13586-bib-0046] Lethin, J. , Shakil, S. S. M. , Hassan, S. , Sirijovski, N. , Töpel, M. , Olsson, O. , & Aronsson, H. (2020). Development and characterization of an EMS‐mutagenized wheat population and identification of salt‐tolerant wheat lines. BMC Plant Biology, 20(1), 1–15. 10.1186/S12870-019-2137-8/FIGURES/11 31931695 PMC6958588

[eva13586-bib-0047] Lin, S. , Cheng, S. , Song, B. , Zhong, X. , Lin, X. , Li, W. , Li, L. , Zhang, Y. , Zhang, H. , Ji, Z. , Cai, M. , Zhuang, Y. , Shi, X. , Lin, L. , Wang, L. , Wang, Z. , Liu, X. , Yu, S. , Zeng, P. , … Morse, D. (2015). The *Symbiodinium kawagutii* genome illuminates dinoflagellate gene expression and coral symbiosis. Science, 350(6261), 691–694. 10.1126/science.aac8370 26542574

[eva13586-bib-0048] Little, A. F. , Van Oppen, M. J. H. , & Willis, B. L. (2004). Flexibility in algal endosymbioses shapes growth in reef corals. Science, 304(5676), 1492–1494. 10.1126/science.1095733 15178799

[eva13586-bib-0049] Littman, R. A. , van Oppen, M. J. H. , & Willis, B. L. (2008). Methods for sampling free‐living *Symbiodinium* (zooxanthellae) and their distribution and abundance at Lizard Island (Great Barrier Reef). Journal of Experimental Marine Biology and Ecology, 364(1), 48–53. 10.1016/j.jembe.2008.06.034

[eva13586-bib-0050] Matsuda, S. B. , Chakravarti, L. J. , Cunning, R. , Huffmyer, A. S. , Nelson, C. E. , Gates, R. D. , & Oppen, M. J. H. (2022). Temperature‐mediated acquisition of rare heterologous symbionts promotes survival of coral larvae under ocean warming. Global Change Biology, 28(6), 2006–2025. 10.1111/gcb.16057 34957651 PMC9303745

[eva13586-bib-0051] Matsuda, S. B. , Huffmyer, A. S. , Lenz, E. A. , Davidson, J. M. , Hancock, J. R. , Przybylowski, A. , Innis, T. , Gates, R. D. , & Barott, K. L. (2020). Coral bleaching susceptibility is predictive of subsequent mortality within but not between coral species. Frontiers in Ecology and Evolution, 8, 178. 10.3389/fevo.2020.00178

[eva13586-bib-0052] McCallum, C. M. , Comai, L. , Greene, E. A. , & Henikoff, S. (2000). Targeted screening for induced mutations. Nature Biotechnology, 18(4), 455–457. 10.1038/74542 10748531

[eva13586-bib-0053] McIlroy, S. E. , Wong, J. C. Y. , & Baker, D. M. (2020). Competitive traits of coral symbionts may alter the structure and function of the microbiome. ISME Journal, 14(10), 2424–2432. 10.1038/s41396-020-0697-0 32518247 PMC7490369

[eva13586-bib-0054] Mieog, J. C. , Olsen, J. L. , Berkelmans, R. , Bleuler‐Martinez, S. A. , Willis, B. L. , & van Oppen, M. J. H. (2009). The roles and interactions of symbiont, host and environment in defining coral fitness. PLoS One, 4(7), e6364. 10.1371/journal.pone.0006364 19629182 PMC2710517

[eva13586-bib-0055] Morris, L. A. , Voolstra, C. R. , Quigley, K. M. , Bourne, D. G. , & Bay, L. K. (2019). Nutrient availability and metabolism affect the stability of coral–Symbiodiniaceae symbioses. Trends in Microbiology, 27(8), 678–689. 10.1016/j.tim.2019.03.004 30987816

[eva13586-bib-0056] Ong, S. C. , Kao, C. Y. , Chiu, S. Y. , Tsai, M. T. , & Lin, C. S. (2010). Characterization of the thermal‐tolerant mutants of *Chlorella* sp. with high growth rate and application in outdoor photobioreactor cultivation. Bioresource Technology, 101(8), 2880–2883. 10.1016/j.biortech.2009.10.007 19897359

[eva13586-bib-0057] Orefice, I. , Musella, M. , Smerilli, A. , Sansone, C. , Chandrasekaran, R. , Corato, F. , & Brunet, C. (2019). Role of nutrient concentrations and water movement on diatom's productivity in culture. Scientific Reports, 9(1), 1479. 10.1038/s41598-018-37611-6 30728371 PMC6365584

[eva13586-bib-0058] Pernice, M. , Meibom, A. , van den Heuvel, A. , Kopp, C. , Domart‐Coulon, I. , Hoegh‐Guldberg, O. , & Dove, S. (2012). A single‐cell view of ammonium assimilation in coral‐dinoflagellate symbiosis. ISME Journal, 6(7), 1314–1324. 10.1038/ismej.2011.196 22222466 PMC3379633

[eva13586-bib-0059] Pochon, X. , & LaJeunesse, T. C. (2021). *Miliolidium* n. gen, a new Symbiodiniacean genus whose members associate with *Soritid foraminifera* or are free‐living. Journal of Eukaryotic Microbiology, 68(4), e12856. 10.1111/jeu.12856 33966311

[eva13586-bib-0060] Qin, Z. , Yu, K. , Chen, B. , Wang, Y. , Liang, J. , Luo, W. , Xu, L. , & Huang, X. (2019). Diversity of Symbiodiniaceae in 15 coral species from the southern South China sea: Potential relationship with coral thermal adaptability. Frontiers in Microbiology, 10, 2343. 10.3389/fmicb.2019.02343 31681208 PMC6813740

[eva13586-bib-0061] Quigley, K. M. , & van Oppen, M. J. H. (2022). Predictive models for the selection of thermally tolerant corals based on offspring survival. Nature Communications, 13(1), 1543. 10.1038/s41467-022-28956-8 PMC8964693 35351901

[eva13586-bib-0062] Quigley, K. M. , Willis, B. L. , & Bay, L. K. (2016). Maternal effects and *Symbiodinium* community composition drive differential patterns in juvenile survival in the coral *Acropora tenuis* . Royal Society Open Science, 3(10), 160471. 10.1098/rsos.160471 27853562 PMC5098987

[eva13586-bib-0063] Rädecker, N. , Pogoreutz, C. , Gegner, H. M. , Cárdenas, A. , Roth, F. , Bougoure, J. , Guagliardo, P. , Wild, C. , Pernice, M. , Raina, J.‐B. , Meibom, A. , & Voolstra, C. R. (2021). Heat stress destabilizes symbiotic nutrient cycling in corals. PNAS, 118(5), e2022653118. 10.1073/pnas.2022653118/-/DCSupplemental 33500354 PMC7865147

[eva13586-bib-0064] Reynolds, J. M. , Bruns, B. U. , Fitt, W. K. , & Schmidt, G. W. (2008). Enhanced photoprotection pathways in symbiotic dinoflagellates of shallow‐water corals and other cnidarians. Proceedings of the National Academy of Sciences of the United States of America, 105(36), 13674–13678. 10.1073/pnas.0805187105 18757737 PMC2527352

[eva13586-bib-0065] Sachdeva, N. , Gupta, R. P. , Mathur, A. S. , & Tuli, D. K. (2016). Enhanced lipid production in thermo‐tolerant mutants of *Chlorella pyrenoidosa* NCIM 2738. Bioresource Technology, 221, 576–587. 10.1016/j.biortech.2016.09.049 27689351

[eva13586-bib-0066] Scharfenstein, H. J. , Chan, W. Y. , Buerger, P. , Humphrey, C. , & van Oppen, M. J. H. (2022). Evidence for de novo acquisition of microalgal symbionts by bleached adult corals. The ISME Journal, 16, 1676–1679. 10.1038/s41396-022-01203-0 35132118 PMC9122906

[eva13586-bib-0067] Schaum, C. E. , Buckling, A. , Smirnoff, N. , Studholme, D. J. , & Yvon‐Durocher, G. (2018). Environmental fluctuations accelerate molecular evolution of thermal tolerance in a marine diatom. Nature Communications, 9(1), 1–14. 10.1038/s41467-018-03906-5 PMC5928086 29712900

[eva13586-bib-0068] Silverstein, R. N. , Correa, A. M. S. , & Baker, A. C. (2012). Specificity is rarely absolute in coral–algal symbiosis: Implications for coral response to climate change. Proceedings of the Royal Society B: Biological Sciences, 279(1738), 2609–2618. 10.1098/rspb.2012.0055 PMC3350700 22367985

[eva13586-bib-0069] Silverstein, R. N. , Cunning, R. , & Baker, A. C. (2015). Change in algal symbiont communities after bleaching, not prior heat exposure, increases heat tolerance of reef corals. Global Change Biology, 21(1), 236–249. 10.1111/gcb.12706 25099991

[eva13586-bib-0070] Silverstein, R. N. , Cunning, R. , & Baker, A. C. (2017). Tenacious D: *Symbiodinium* in clade D remain in reef corals at both high and low temperature extremes despite impairment. Journal of Experimental Biology, 220(7), 1192–1196. 10.1242/jeb.148239 28108671

[eva13586-bib-0071] Smith, E. G. , Ketchum, R. N. , & Burt, J. A. (2017). Host specificity of *Symbiodinium* variants revealed by an ITS2 metahaplotype approach. ISME Journal, 11(6), 1500–1503. 10.1038/ismej.2016.206 28211848 PMC5437344

[eva13586-bib-0072] Spalding, M. D. , & Brown, B. E. (2015). Warm‐water coral reefs and climate change. Science, 350(6262), 769–771. 10.1126/science.aad0349 26564846

[eva13586-bib-0073] Stat, M. , & Gates, R. D. (2008). Vectored introductions of marine endosymbiotic dinoflagellates into Hawaii. Biological Invasions, 10(4), 579–583. 10.1007/s10530-007-9167-0

[eva13586-bib-0074] Stearns, S. C. (1989). Trade‐offs in life‐history evolution. Functional Ecology, 3(3), 259. 10.2307/2389364

[eva13586-bib-0075] Suggett, D. J. , Goyen, S. , Evenhuis, C. , Szabó, M. , Pettay, D. T. , Warner, M. E. , & Ralph, P. J. (2015). Functional diversity of photobiological traits within the genus *Symbiodinium* appears to be governed by the interaction of cell size with cladal designation. New Phytologist, 208(2), 370–381. 10.1111/nph.13483 26017701

[eva13586-bib-0076] Suzuki, G. , Yamashita, H. , Kai, S. , Hayashibara, T. , Suzuki, K. , Iehisa, Y. , Okada, W. , Ando, W. , & Komori, T. (2013). Early uptake of specific symbionts enhances the post‐settlement survival of *Acropora* corals. Marine Ecology Progress Series, 494, 149–158. 10.3354/meps10548

[eva13586-bib-0077] Teschima, M. M. , Garrido, A. , Paris, A. , Nunes, F. L. D. , & Zilberberg, C. (2019). Biogeography of the endosymbiotic dinoflagellates (Symbiodiniaceae) community associated with the brooding coral *Favia gravida* in the Atlantic Ocean. PLoS One, 14(3), e0213519. 10.1371/journal.pone.0213519 30849101 PMC6407780

[eva13586-bib-0078] Thomas, L. , Kendrick, G. A. , Kennington, W. J. , Richards, Z. T. , & Stat, M. (2014). Exploring *Symbiodinium* diversity and host specificity in *Acropora* corals from geographical extremes of Western Australia with 454 amplicon pyrosequencing. Molecular Ecology, 23(12), 3113–3126. 10.1111/mec.12801 24845644

[eva13586-bib-0079] Tillich, U. M. , Lehmann, S. , Schulze, K. , Dühring, U. , & Frohme, M. (2012). The optimal mutagen dosage to induce point‐mutations in *Synechocystis* sp. PCC6803 and its application to promote temperature tolerance. PLoS ONE, 7(11), e49467. 10.1371/journal.pone.0049467 23185339 PMC3504032

[eva13586-bib-0080] van Oppen, M. J. H. , Gates, R. D. , Blackall, L. L. , Cantin, N. , Chakravarti, L. J. , Chan, W. Y. , Cormick, C. , Crean, A. , Damjanovic, K. , Epstein, H. , Harrison, P. L. , Jones, T. A. , Miller, M. , Pears, R. J. , Peplow, L. M. , Raftos, D. A. , Schaffelke, B. , Stewart, K. , Torda, G. , … Putnam, H. M. (2017). Shifting paradigms in restoration of the world's coral reefs. Global Change Biology, 23(9), 3437–3448. 10.1111/gcb.13647 28247459

[eva13586-bib-0081] van Oppen, M. J. H. , Oliver, J. K. , Putnam, H. M. , & Gates, R. D. (2015). Building coral reef resilience through assisted evolution. Proceedings of the National Academy of Sciences of the United States of America, 112(8), 2307–2313. 10.1073/pnas.1422301112 25646461 PMC4345611

[eva13586-bib-0082] Warner, M. E. , Fitt, W. K. , & Schmidt, G. W. (1999). Damage to photosystem II in symbiotic dinoflagellates: A determinant of coral bleaching. PNAS, 96(14), 8007–8012. 10.1073/pnas.96.14.8007 10393938 PMC22178

[eva13586-bib-0083] Weber, M. X. , & Medina, M. (2012). The role of microalgal symbionts (*Symbiodinium*) in Holobiont physiology. Advances in Botanical Research, 64, 119–140. 10.1016/B978-0-12-391499-6.00004-9

[eva13586-bib-0084] Weis, V. M. (2008). Cellular mechanisms of cnidarian bleaching: Stress causes the collapse of symbiosis. Journal of Experimental Biology, 211(19), 3059–3066. 10.1242/jeb.009597 18805804

[eva13586-bib-0085] Wiedenmann, J. , D'Angelo, C. , Smith, E. G. , Hunt, A. N. , Legiret, F. E. , Postle, A. D. , & Achterberg, E. P. (2013). Nutrient enrichment can increase the susceptibility of reef corals to bleaching. Nature Climate Change, 3(2), 160–164. 10.1038/nclimate1661

[eva13586-bib-0086] Wilkerson, F. P. , Kobayashi, D. , & Muscatine, L. (1988). Mitotic index and size of symbiotic algae in Caribbean reef corals. Coral Reefs, 7(1), 29–36. 10.1007/BF00301979

[eva13586-bib-0087] Wong, J. C. Y. , Enríquez, S. , & Baker, D. M. (2021). Towards a trait‐based understanding of Symbiodiniaceae nutrient acquisition strategies. Coral Reefs, 40(2), 625–639. 10.1007/s00338-020-02034-1

[eva13586-bib-0088] Wooldridge, S. A. (2013). Breakdown of the coral‐algae symbiosis: Towards formalising a linkage between warm‐water bleaching thresholds and the growth rate of the intracellular zooxanthellae. Biogeosciences, 10(3), 1647–1658. 10.5194/bg-10-1647-2013

[eva13586-bib-0089] Ziegler, M. , Eguíluz, V. M. , Duarte, C. M. , & Voolstra, C. R. (2018). Rare symbionts may contribute to the resilience of coral‐algal assemblages. ISME Journal, 12(1), 161–172. 10.1038/ismej.2017.151 29192903 PMC5739009

