# Supplementary material for: Chemical mutagenesis and thermal selection of coral photosymbionts induce adaptation to heat stress with trait trade‐offs
Source: Evol Appl. 2023 Aug 19;16(9):1549–67. doi: 10.1111/eva.13586 (PMC10519419; doi:10.1111/eva.13586)
Supplement: Supplementary file 1 — Data S1 [file EVA-16-1549-s001.docx]

**Supplementary Tables and Figures**

**Table S1: Recipe for Daigo’s IMK culture medium used for Symbiodiniaceae cultivation.** Actual macronutrient concentration measured in a fresh preparation of IMK:

NO_3_^-^ = 2526 μM; NH_4_^+^ = 63 μM; PO_4_^-3^ = 45 μM.

|  | **Daigos' IMK recipe** |
| --- | --- |
| **Water base (MilliQ)** | 1 L |
| **Macronutrients (μM)** |  |
| NaNO_3_ | 2353 |
| NH_4_Cl | 50 |
| Na_2_HPO_4_ | 9.9 |
| K_2_HPO_4_ | 28.7 |
| Na_2_EDTA | 110.6 |
| Fe-EDTA | 14.2 |
| Mn-EDTA | 0.96 |
| **Trace Metals (μM)** |  |
| CuSO_4.5_H_2_O | 0.01 |
| ZnSO_4_._7_H_2_O | 0.08 |
| MnCl_2_._4_H_2_O | 0.91 |
| Na_2_MoO_4.2_H_2_O | 0.03 |
| CoSO_4_._7_H_2_O | 0.05 |
| H_2_SeO_3_ | 0.01 |
| **Vitamins (μM)** |  |
| Biotin | 0.0061 |
| Vitamin B_12_ | 0.0011 |
| Thiamine HCl | 0.593 |

**Table S2:** **Summary of pairwise comparisons of the estimated marginal means calculated for each physiological trait**. Pairwise comparisons were carried out between strains (strain contrast: WT/MT) at each temperature treatment and for each strain between both temperatures (temperature contrast: Ambient/Elevated) at each experimental phase. For the intracellular ROS (InROS) and extracellular ROS (ExROS) analyses, pairwise comparisons (WT-MT) were carried out between strains at each timepoint (t1-4), rather than experimental phase.

| **Response** | **Contrast** | **Level** | **Experimental phase** | ***D. trenchii*** | | ***F. kawagutii*** | | ***S. pilosum*** | |
| --- | --- | --- | --- | --- | --- | --- | --- | --- | --- |
|  |  |  |  | **t-value** | **p-value** | **t-value** | **p-value** | **t-value** | **p-value** |
| Cell density yields | Strain contrast (WT vs. MT) | Ambient | Early | -0.9 | 0.378 | -2.1 | 0.034 | -1.0 | 0.305 |
|  |  |  | Mid | 9.1 | < 0.001 | -1.6 | 0.120 | 2.9 | 0.005 |
|  |  |  | Late | 14.9 | < 0.001 | 1.0 | 0.338 | -2.0 | 0.046 |
|  |  | Elevated | Early | -5.6 | < 0.001 | 1.1 | 0.276 | -2.5 | 0.015 |
|  |  |  | Mid | 2.2 | 0.032 | 2.8 | 0.006 | -3.4 | < 0.001 |
|  |  |  | Late | 8.6 | < 0.001 | -3.7 | < 0.001 | -17.4 | < 0.001 |
|  | Temperature contrast (Ambient vs. Elevated) | WT | Early | 13.0 | < 0.001 | -5.2 | < 0.001 | 0.9 | 0.369 |
|  |  |  | Mid | 28.5 | < 0.001 | -4.4 | < 0.001 | 14.6 | < 0.001 |
|  |  |  | Late | 34.7 | < 0.001 | -2.9 | 0.005 | 44.9 | < 0.001 |
|  |  | MT | Early | 8.3 | < 0.001 | -2.0 | 0.052 | -0.5 | 0.604 |
|  |  |  | Mid | 21.5 | < 0.001 | 0.0 | 0.987 | 8.3 | < 0.001 |
|  |  |  | Late | 28.4 | < 0.001 | -7.6 | < 0.001 | 29.5 | < 0.001 |
| Growth rates | Strain contrast (WT vs. MT) | Ambient | / | 11.8 | < 0.001 | -0.5 | 0.595 | -0.3 | 0.794 |
|  |  | Elevated |  | 6.8 | < 0.001 | 0.6 | 0.544 | -0.4 | 0.705 |
|  | Temperature contrast (Ambient vs. Elevated) | WT | / | 19.8 | < 0.001 | -2.5 | 0.025 | 6.7 | < 0.001 |
|  |  | MT |  | 14.8 | < 0.001 | -1.3 | 0.207 | 6.6 | < 0.001 |
| F_v_/F_m_ | Strain contrast (WT vs. MT) | Ambient | Early | 12.0 | < 0.001 | -0.8 | 0.398 | 3.4 | < 0.001 |
|  |  |  | Mid | 5.9 | < 0.001 | -1.3 | 0.185 | 1.3 | 0.205 |
|  |  |  | Late | 5.8 | < 0.001 | 1.5 | 0.141 | 6.3 | < 0.001 |
|  |  | Elevated | Early | -7.5 | < 0.001 | 0.3 | 0.742 | 14.9 | < 0.001 |
|  |  |  | Mid | -11.7 | < 0.001 | -0.4 | 0.672 | 3.0 | 0.003 |
|  |  |  | Late | -10.9 | < 0.001 | -15.5 | < 0.001 | -24.0 | < 0.001 |
|  | Temperature contrast (Ambient vs. Elevated) | WT | Early | 6.9 | < 0.001 | 17.6 | < 0.001 | 12.2 | < 0.001 |
|  |  |  | Mid | 19.6 | < 0.001 | 37.7 | < 0.001 | 23.5 | < 0.001 |
|  |  |  | Late | 26.9 | < 0.001 | 54.8 | < 0.001 | 50.9 | < 0.001 |
|  |  | MT | Early | -12.6 | < 0.001 | 18.8 | < 0.001 | 23.6 | < 0.001 |
|  |  |  | Mid | 2.1 | 0.036 | 38.6 | < 0.001 | 27.7 | < 0.001 |
|  |  |  | Late | 10.3 | < 0.001 | 41.6 | < 0.001 | 22.1 | < 0.001 |
| Q_m_ | Strain contrast (WT vs. MT) | Ambient | Early | -4.2 | < 0.001 | 4.4 | < 0.001 | -2.7 | 0.006 |
|  |  |  | Mid | -5.2 | < 0.001 | 6.8 | < 0.001 | -4.8 | < 0.001 |
|  |  |  | Late | -0.8 | 0.405 | 8.5 | < 0.001 | -2.6 | 0.010 |
|  |  | Elevated | Early | -5.6 | < 0.001 | -1.2 | 0.240 | -2.0 | 0.050 |
|  |  |  | Mid | 5.5 | < 0.001 | -0.7 | 0.460 | -3.6 | < 0.001 |
|  |  |  | Late | 6.7 | < 0.001 | -4.5 | < 0.001 | 5.4 | < 0.001 |
|  | Temperature contrast (Ambient vs. Elevated) | WT | Early | 0.4 | 0.659 | -3.4 | < 0.001 | 3.7 | < 0.001 |
|  |  |  | Mid | -2.3 | 0.023 | -10.7 | < 0.001 | -7.8 | < 0.001 |
|  |  |  | Late | -5.6 | < 0.001 | -5.9 | < 0.001 | -17.1 | < 0.001 |
|  |  | MT | Early | -0.9 | 0.346 | -8.9 | < 0.001 | 6.1 | < 0.001 |
|  |  |  | Mid | 8.3 | < 0.001 | -17.4 | < 0.001 | -6.8 | < 0.001 |
|  |  |  | Late | 2.0 | 0.046 | -18.0 | < 0.001 | -11.1 | < 0.001 |
| InROS levels | Strain contrast (WT vs. MT) | / | t1 | -2.7 | 0.012 | -0.7 | 0.517 | 0.2 | 0.854 |
|  |  |  | t2 | -4.1 | < 0.001 | -1.0 | 0.343 | 1.0 | 0.338 |
|  |  |  | t3 | -1.7 | 0.106 | 0.7 | 0.472 | 0.5 | 0.629 |
|  |  |  | t4 | 0.1 | 0.934 | 0.9 | 0.381 | -0.2 | 0.853 |
| ExROS levels | Strain contrast (WT vs. MT) | / | t1 | -5.5 | < 0.001 | 0.3 | 0.790 | -1.6 | 0.113 |
|  |  |  | t2 | 3.5 | 0.001 | -1.0 | 0.338 | 1.3 | 0.196 |
|  |  |  | t3 | -1.3 | 0.211 | -0.1 | 0.943 | 1.9 | 0.066 |
|  |  |  | t4 | -2.3 | 0.026 | 1.4 | 0.163 | 5.7 | < 0.001 |

**Figure S1: Experimental designs of experimental evolution (A) and reciprocal transplant (B) experiments.** For clarity, the reciprocal transplant experiment (RTE) is illustrated with *D. trenchii* only. An identical design was applied for *F. kawagutii* and *S. pilosum*, with the exception of the elevated temperature being set at 35°C. During the RTE, total replication per strain at each temperature was 50.


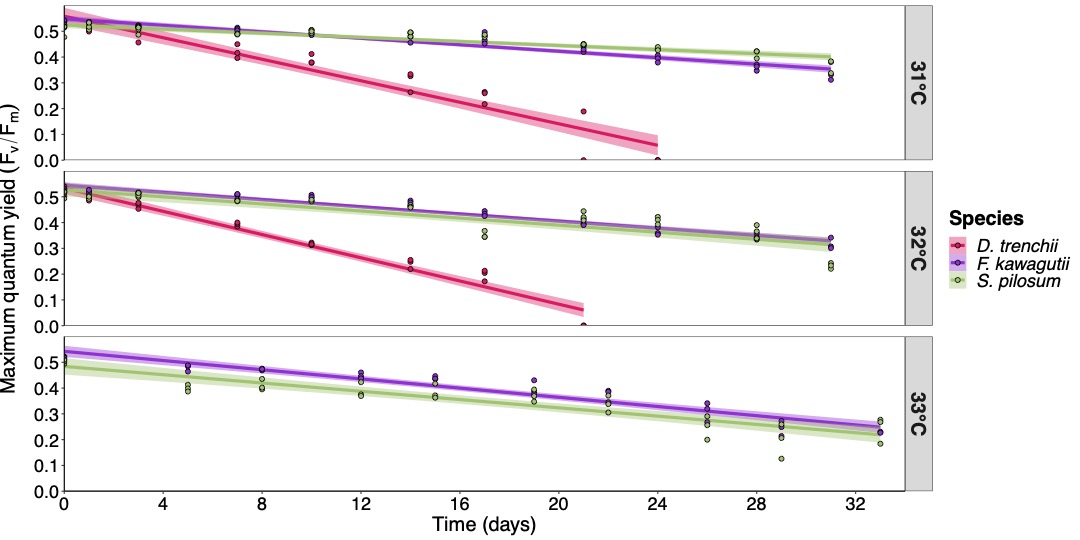


**Figure S2: Photochemical efficiency** **of *Durusdinium trenchii* (SCF086.01)*, Fugacium kawagutii* (SCF089.01) and *Symbiodinium pilosum* (SCF004.01) strains at 31-33°C.** Assay was carried out during June 2016 , prior to experimental evolution. Temperatures identified to induce the greatest decreases in F_v_/F_m_ (i.e., greatest negative deltas between start and end F_v_/F_m_) were used as temperature for thermal selection during experimental evolution. For *D. trenchii* this corresponded to 31/32°C, whilst for *F. kawagutii* and *S. pilosum* 33/34°C were selected (exposure to 33°C did not cause cultures to fully crash, hence a higher temperature was included).


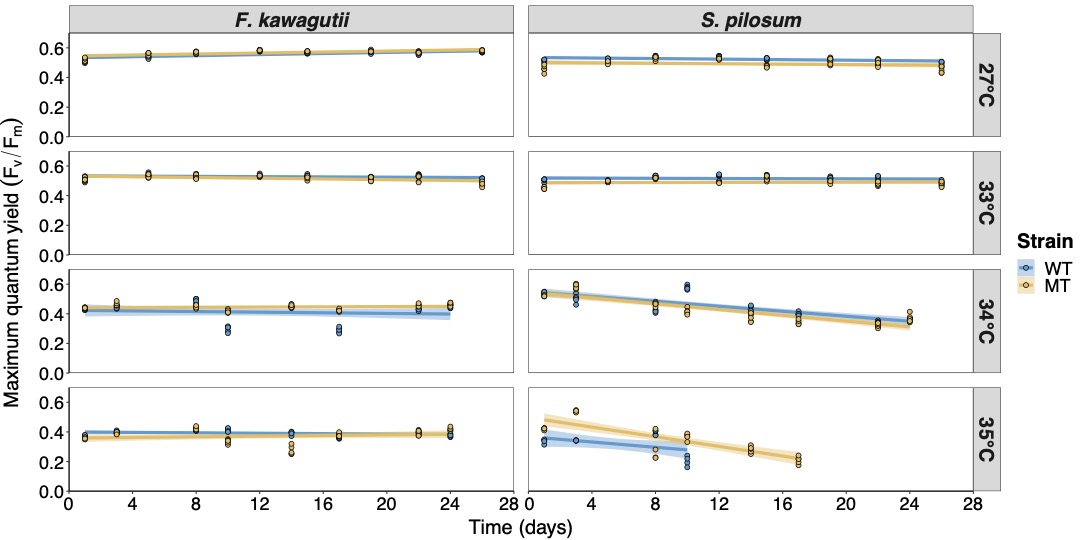


**Figure S3: Photochemical efficiency of *Fugacium kawagutii* and *Symbiodinium pilosum* lineages.** Mean maximum quantum yields of mutagen-treated (MT) and wild-type (WT) strains at ambient (27°C) and elevated (33-35°C) temperatures. The assay was carried out in September 2021, prior to the reciprocal transplant experiment of *F. kawagutii* and *S. pilosum*. The temperature which led to the greatest decrease in F_v_/F_m_ relative to F_v_/F_m_ at ambient temperature (27°C) was selected as the elevated temperature for the reciprocal transplant experiment (in this case 35°C). n=5 for each timepoint.

**Figure S4**: **Physiological performances of *Durusdinium trenchii* cultures.** Mean cell densities and maximum quantum yields (**A**), intracellular and extracellular ROS levels (**B**) of mutagen-treated (MT) and wild-type (WT) strains at ambient (27°C) and elevated (35°C) temperatures. Intracellular ROS measurements were obtained from the median fluorescent intensities of Symbiodiniaceae cells stained with CellROX Green. Extracellular ROS values were obtained from the absorbance of cells stained with CellROX Orange and normalised to the cell density measured from the same cultures. Error bars represent 1 standard error (**A**); n=5 for each timepoint.

**Figure S5**: **Physiological performances of *Fugacium kawagutii* cultures.** Mean cell densities and maximum quantum yields (**A**), intracellular and extracellular ROS levels (**B**) of mutagen-treated (MT) and wild-type (WT) strains at ambient (27°C) and elevated (35°C) temperatures. Intracellular ROS measurements were obtained from the median fluorescent intensities of Symbiodiniaceae cells stained with CellROX Green. Extracellular ROS values were obtained from the absorbance of cells stained with CellROX Orange and normalised to the cell density measured from the same cultures. Error bars represent 1 standard error (**A**); n=5 for each timepoint.

**Figure S6**: **Physiological performances of *Symbiodinium pilosum* cultures.** Mean cell densities and maximum quantum yields (**A**), intracellular and extracellular ROS levels (**B**) of mutagen-treated (MT) and wild-type (WT) strains at ambient (27°C) and elevated (35°C) temperatures. Intracellular ROS measurements were obtained from the median fluorescent intensities of Symbiodiniaceae cells stained with CellROX Green. Extracellular ROS values were obtained from the absorbance of cells stained with CellROX Orange and normalised to the cell density measured from the same cultures. Error bars represent 1 standard error (**A**); n=5 for each timepoint.
